# Fine‐Scale Spatial Genetic Structure and Leaf Shape Variation in Five Fagaceae Species: Insights Into Conservation and Adaptation

**DOI:** 10.1002/ece3.72863

**Published:** 2026-02-18

**Authors:** Rongle Wang, Yanjun Luo, Min Qi, Yi Zhang, Jiawen Zhang, Yibo Luo, Fang K. Du

**Affiliations:** ^1^ School of Ecology and Nature Conservation Beijing Forestry University Beijing China; ^2^ State Key Laboratory of Plant Diversity and Specialty Crops, Institute of Botany, Chinese Academy of Sciences Beijing China; ^3^ Department of Forest Molecular Genetics and Biotechnology Forestry and Forest Products Research Institute, Forest Research and Management Organization Ibaraki Japan

**Keywords:** *Castanopsis*, fine‐scale spatial genetic structure, leaf morphology, *Quercus*, species conservation

## Abstract

Fine‐scale spatial genetic structure (fine‐scale SGS) refers to the pattern of spatial distribution of genetic variation at the local scale, which can indirectly estimate gene flow among individuals and reveal microevolutionary processes in plant populations. Although fine‐scale SGS is important in explaining dispersal patterns and adaptive variation in plants, few studies have explored its potential application in species conservation strategies. In addition, phenotypic traits, particularly leaf shape, may also exhibit specific spatial variation patterns at fine scales. In this study, we investigated the genetic and leaf shape variation of two genus *Quercus* species ( *Quercus glauca* Thunb. and *Q*. *multinervis* J. Q. Li) and three genus *Castanopsis* species (*Castanopsis tibetana* Hance, *C*. *faberi* Hance, and *C. fargesii* Franch.) in Wuyishan National Park in southeastern China. Using genetic markers, we found that *Quercus* species exhibited stronger fine‐scale SGS and more limited gene flow than *Castanopsis* species, suggesting greater habitat fragmentation affecting local *Quercus* species. Leaf morphological analysis revealed inter‐generic differences and partial overlap in leaf shape between *Quercus* and *Castanopsis* species, with the greatest variation observed in leaf area (LA) and leaf mass (LM). In addition, all five Fagaceae species exhibited significant diminishing returns, with *C. fargesii* showing the most pronounced effect and possessing the smallest leaves, which may enhance its adaptability to the harsh environments. Despite the leaf shape overlaps blurring species boundaries between *Quercus* and *Castanopsis* species, their genetic structure is remained clearly distinct. The observed differences in the intensity of fine‐scale SGS and leaf shape variation between the two genera reflect their different environmental adaptability, offering new insights into the integration of genetic and phenotypic data for conservation planning.

## Graphical abstract

We investigated both fine‐scale SGS and leaf shape variation in five Fagaceae species ( *Q. glauca* , *Q. multinervis*, *C. tibetana* , *C. faberi*, and *C. fargesii* ) from the genera *Quercus* and *Castanopsis* in Wuyishan National Park. We found that *Quercus* species exhibit stronger fine‐scale SGS than *Castanopsis* species. These two genera show differences in leaf shape, and all five species exhibit a pattern of diminishing returns in leaf trait scaling. Our findings underscore the importance of conserving fine‐scale SGS and considering leaf shape variation in the design of effective conservation strategies.

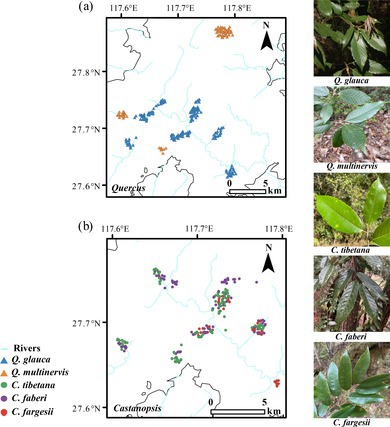

## 1 Introduction

Fine‐scale spatial genetic structure (fine‐scale SGS) refers to the non‐random spatial distribution of genotypes at the local scale, shaped primarily by limited gene flow (Gamba and Muchhala 2023; Vekemans and Hardy 2004). By quantifying genetic similarity in relation to spatial distance among individuals, researchers can effectively detect patterns of spatial genetic structure and evaluate its intensity, thereby gaining insights into the dispersal patterns of both pollen and seeds (Angbonda et al. 2021).

Fine‐scale SGS is influenced by many factors, including limited gene flow, population density, habitat fragmentation, genetic drift, and microhabitat selection (Wang et al. 2017). In plants, gene flow can be mediated by pollen and seed, plays a central role in shaping patterns of fine‐scale SGS. In particular, pollen‐mediated gene flow is particularly important because it influences the effective reproductive range, enhances population connectivity, and influences the extent to which habitat fragmentation affects genetic structure (Ashley 2010). Restricted pollen dispersal can increase the intensity of fine‐scale SGS. Likewise, limited seed dispersal reinforces these patterns by promoting the spatial clustering of genetically related individuals. In tree species, seeds tend to fall close to the maternal tree, leading to spatial clustering of related individuals (Berg and Hamrick 1994). Such localized seed dispersal, characterized by short dispersal distances, reinforces this clustering pattern and consequently promotes strong fine‐scale SGS within populations (Buzatti et al. 2012).

In addition to genetic factors, phenotypic traits such as leaf shape may also exhibit specific distribution patterns at a fine spatial scale. Leaf shape, closely linked to functional traits, serves as a key feature in plants and plays an important role in plant taxonomy and systematics and also reflects adaptive response to environmental changes (Du et al. 2022; Nicotra et al. 2011; Qi et al. 2025; Ramírez‐Valiente et al. 2017; Zhang et al. 2025). Meanwhile, genetic structure at a fine spatial scale can influence local adaptation by shaping patterns of gene flow, genetic drift, and selection, thereby affecting the evolutionary potential of populations. Therefore, understanding fine‐scale SGS and phenotype variation, such as leaf shape, can reveal how evolutionary processes act on both genetic and phenotypic levels to drive plant adaptation. Integrating fine‐scale SGS with analyses of leaf shape variation helps identify whether similar phenotypes arise from shared genetic backgrounds or represent independent local adaptations, offering a more comprehensive view of adaptation at a fine spatial scale.

In studies of leaf shape, traditional morphometric methods are commonly used to quantify variation through multivariate statistical analysis of quantitative and qualitative leaf traits, such as the linear distance, angle, and area (Henderson 2006). Specific leaf area (SLA), as a comprehensive indirect leaf trait, represents the trade‐off between leaf mass and leaf size (Pérez‐Harguindeguy et al. 2016). However, this trade‐off is not always straightforward; sometimes the leaf area may fail to keep pace with the increase in leaf dry mass, a phenomenon referred to as “diminishing returns” (Niklas et al. 2007, 2009). However, traditional morphometric methods studies are often influenced by leaf size and may not effectively capture leaf shape variation in an intuitive way. Current developed geometric morphometric methods (GMMs) can digitize leaf shape based on Cartesian landmark coordinates, using multivariate statistical analysis to identify leaf shape variation among species to avoid interference (such as leaf size, direction, and location), and can directly reflect leaf shape variation (Du et al. 2022; Klingenberg 2011; Ray 1992; Viscosi and Cardini 2011).

Fagaceae (ca. 1000 species across eight genera) is one of the most important families of temperate and subtropical forests worldwide (Govaerts et al. 1998). In China, Fagaceae species inhabit a wide range of environments, from the Himalayas in the west to Taiwan Island in the east (Huang et al. 1999). Many studies indicate that interspecific hybridization was prevalent in Fagaceae species, such as between *Quercus rubra* and *Q. ellipsoidalis* (Gailing et al. 2012), *Q*. *acutifolia* and *Q*. *grahamii* (Pérez‐Pedraza et al. 2021), *Castanopsis sieboldii* and *C. cuspidata* (Aoki et al. 2014). Hybridization may influence fine‐scale SGS by promoting interspecific gene flow and altering the spatial distribution patterns (Curtu et al. 2015; Valbuena‐Carabaña et al. 2007). Moreover, hybrid individuals may exhibit various intermediate phenotypes (Li et al. 2022). Fagaceae plants often occur sympatrically at a fine spatial scale; therefore, providing a good model to study fine‐scale SGS and leaf shape variation.

Our study focuses on the fine‐scale SGS and leaf shape variation of Fagaceae species located in Wuyishan National Park. Wuyishan National Park represents the most intact and largest mid‐subtropical forest ecosystem in southeastern China (Chen et al. 2024). The area has a typical subtropical monsoon climate with abundant precipitation and full sunshine (Zheng et al. 2025). The average annual precipitation is 1486–2150 mm and the average annual temperature is 8.5°C–18°C. The region offers an ideal habitat and reproductive environment due to its unique topography and habitat diversity (Li et al. 2023). Although long‐term conservation efforts have been implemented, local habitat fragmentation still exists due to extensive plantations of *Phyllostachys edulis* and *Camellia sinensis* (Ding et al. 2015; Yang 2020). Fagaceae species are dominant or constructive components of the local forest, particularly species of genera *Quercus* and *Castanopsis* (Ding et al. 2015). *Quercus*, the largest genus in the Fagaceae family, is known for its high species richness and strong ecological adaptability (Cavender‐Bares 2019; Du et al. 2022; Kremer et al. 2012; Zhang et al. 2025). *Castanopsis*, the third largest genus in the family, consists of dominant trees in subtropical evergreen broad‐leaved forests and tropical seasonal rainforests (Wang et al. 2022). We investigated two *Quercus* species ( *Quercus glauca* Thunb. and *Q*. *multinervis* J. Q. Li) and three *Castanopsis* species (*Castanopsis tibetana* Hance, *C*. *faberi* Hance, and *C. fargesii* Franch.) within Wuyishan National Park. All five tree species are monoecious plants, wind‐pollinated and seeds dispersed by gravity (Chen et al. 2008; Curtu et al. 2015; Liu et al. 2008). Hybridization has been reported between *Q. glauca* and *Q*. *multinervis* (Deng et al. 2013), while no such events have been documented among the other species. The two *Quercus* species differ in leaf morphology; *Q. multinervis* typically has more secondary veins than *Q. glauca* (Huang et al. 1999). In addition, the leaf margin of *Q. glauca* bears sparse serrations above the midpoint, whereas *Q. multinervis* shows sharp serrations restricted to the upper one‐third of the margin (Huang et al. 1999). Among the three *Castanopsis* species, *C. tibetana* has large leaves (c. 15–30 cm long, 5–10 cm wide) with serrate teeth near the apex and a dark brown abaxial surface (Huang et al. 1999). *C. faberi* has leaves with entire margins or a few serrations near the apex, and a compact, grayish‐white layer of scalelike trichomes on the abaxial surface (Huang et al. 1999). *C. fargesii* has leaves with entire margins or a few shallow teeth from middle to apex, and a thick, mealy yellowish‐brown layer of scalelike trichomes on the abaxial surface (Huang et al. 1999). Previous studies have investigated either fine‐scale SGS or leaf shape variation in *Q. glauca* (Gillani et al. 2023; Tong et al. 2021), but few studies have comprehensively explored both fine‐scale SGS and leaf shape variation to analyze these species.

Our study systematically investigates the fine‐scale SGS and leaf shape variation of five evergreen Fagaceae species in Wuyishan National Park by conducting genetic and leaf morphological analyses. In this study, we tested three hypotheses: (1) closely related species exhibit fine‐scale spatial genetic structure due to localized genetic differentiation; (2) leaf morphological traits differ among populations and species as a result of adaptation; and (3) hybridization and gene flow among species partially overlap leaf traits while maintaining distinct genetic boundaries.

## 2 Materials and Methods

### 2.1 Sampling

**FIGURE 1:**
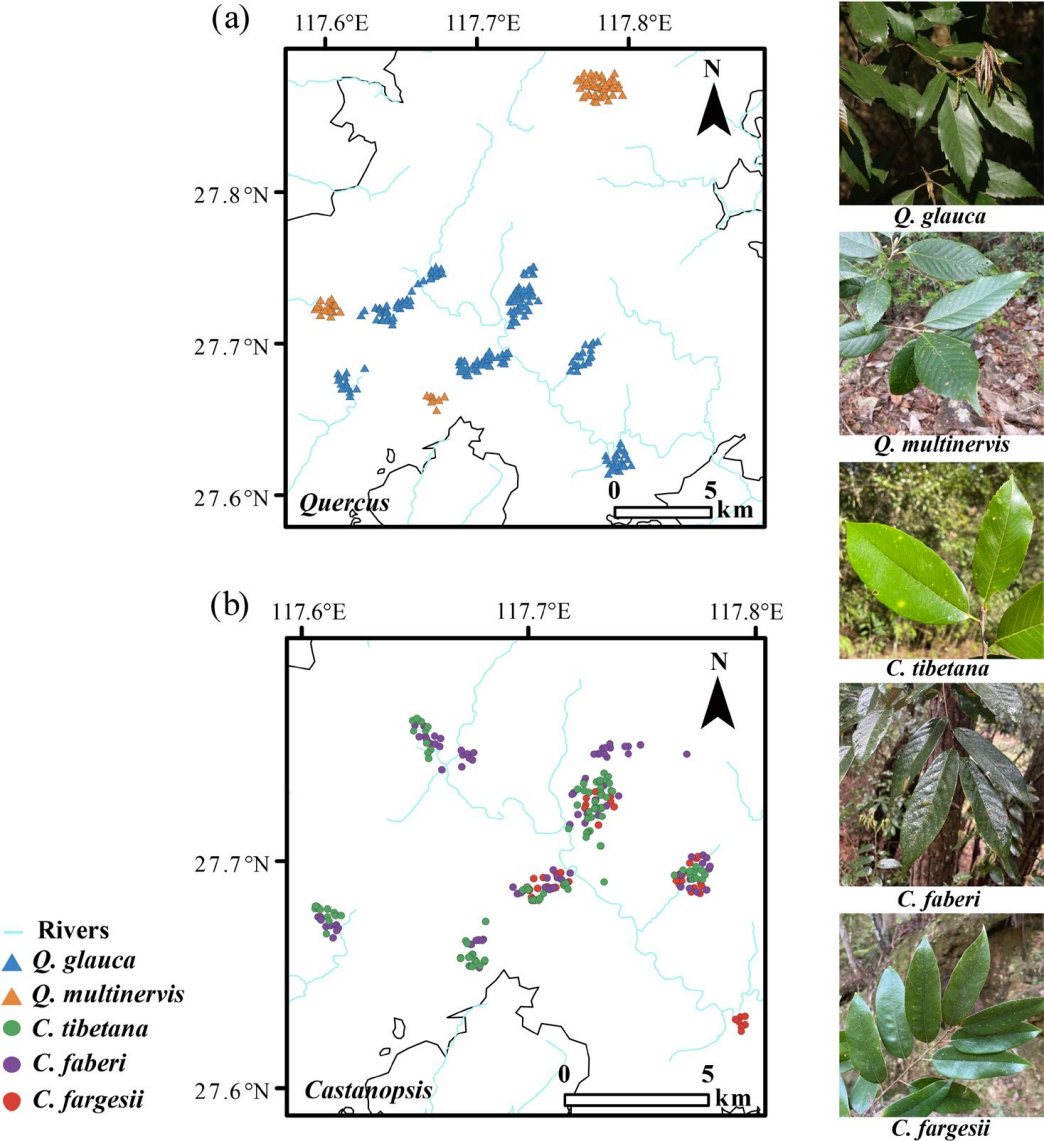
Sampling locations and leaf morphology for five Fagaceae species: *Quercus glauca* , *Q. multinervis*, *Castanopsis tibetana*, *C. faberi* and *C. fargesii* in the Wuyishan National Park. (a) Sampling locations for each individual of the two genus *Quercus* species and (b) three genus *Castanopsis* species. Population codes and site description are given in Table S1. Leaf morphology on the right represents the leaves of *Q. glauca* , *Q. multinervis*, *C. tibetana* , *C. faberi* and *C. fargesii* .

The sampling was conducted in the evergreen broad‐leaved forest at Wuyishan National Park in southeastern China, where Fagaceae is among the most dominant tree species. According to morphological identification, we collected a total of 601 adult trees for five species from the genera *Quercus* and *Castanopsis* of Fagaceae. The sampling covered the entire distribution range of the five study species in Wuyishan National Park. For *Quercus*, we sampled 207 individuals from eight populations of *Q. glauca* and 119 individuals from three populations of *Q. multinervis* (Figure 1a). For *Castanopsis*, we sampled 111 individuals from seven populations of *C. tibetana* , 102 individuals from seven populations of *C. faberi*, and 62 individuals from four populations of *C. fargesii* (Figure 1b). Within each population, each adult tree was sampled at a minimum of 10 m interval (Soto et al. 2007), so as to minimize the sampling of close relatives. We collected a total of five to seven mature and intact leaves along the four cardinal directions in the middle layer of the canopy for leaf morphological analysis, and one to two young leaves or new branches for DNA isolation. We dried all leaf samples in silica gel immediately and recorded the latitude, longitude, and altitude of each individual using a 621sc global positioning system (GPS) device (Garmin, Beijing, China). The detail sampling information was listed in Table S1.

### 2.2 DNA Isolation and Microsatellite Genotyping

We extracted genomic DNA from leaf tissue using a Plant Genomic DNA Extraction Kit (Tiangen, Beijing, China). The DNA quality was initially checked using a 1% agarose gel and then the concentration was measured by an ultramicro‐spectrophotometer (Thermo Fisher, USA). We randomly selected two individuals from each of three distant sites of species for pre‐amplification experiments with 63 nuclear microsatellite (nSSR) loci developed from other Fagaceae species (Table S2), because nSSR offers cost‐effectiveness, reliability, and effectiveness in species identification (Guichoux et al. 2011; Qi et al. 2024). We excluded loci harboring null alleles as identified by MICRO‐CHECKER v.2.2 (Van Oosterhout et al. 2004). We applied 10 successfully amplified nSSR loci for genotyping all 601 individuals (Table S2). These loci, which were previously developed from *Castanea sativa* , *Fagus sylvatica* (Sebastiani et al. 2004), *Q. mongolica* var. *crispula* (Ueno et al. 2008), *Q. robur* , *Q. petraea* (Durand et al. 2010), showed high polymorphism in pre‐amplification experiments. The PCR conditions followed Du et al. (2017) and we analyzed the PCR products using an ABI PRISM 3730 Genetic Analyzer (Applied Biosystems, USA). Subsequently, we scored the alleles using GENEMARKER v.2.2 (Softgenetics, USA) and checked the genotypes twice.

### 2.3 Leaf Morphology

**FIGURE 2:**
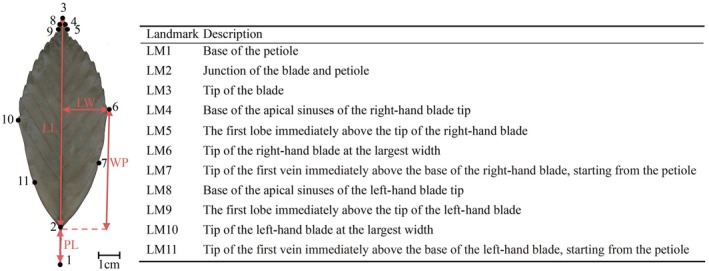
Leaf configuration of *Q. glauca* , showing four traditional leaf morphological traits and locations of the 11 features used as landmarks (LMs) with descriptions of the LMs on the right.

We scanned five intact and dried leaves of each individual with the abaxial surface uppermost using a CanoScan 5600 F scanner (Canon Inc., Japan) at a resolution of 600 dpi. We then conducted traditional morphometric methods and GMMs for leaf shape variation. For traditional morphometric methods, we measured seven leaf traits to study leaf morphological characters, including leaf length (LL, cm), petiole length (PL, cm), leaf width (LW, cm), length of lamina from base to widest point (WP, cm), leaf area (LA, cm^2^), leaf mass (LM, g), and specific leaf area (SLA, cm^2^·g^−1^) (Figure 2). For GMMs, we selected 11 landmarks for each leaf, including three landmarks distributed along the middle axis of the leaf (LM1–LM3) and eight landmarks symmetrically distributed on both sides of the leaf (LM4–LM11) (Figure 2) (Jensen 1990; Savriama and Klingenberg 2011; Viscosi et al. 2009). We organized the raw data for all leaf landmark configurations into 11 pairs of Cartesian coordinates (*x*, *y*) as the core variables using ImageJ v.1.5 (Abràmoff et al. 2004). Then we imported all the *x*, *y* coordinates as input data into MorphoJ for the following analysis (Klingenberg 2011).

### 2.4 Data Analysis

#### 2.4.1 Genetic Diversity and Differentiation

We estimated the number of different alleles (*N*_A_), number of effective alleles (*N*_E_), Shannon's information index (*I*), observed heterozygosity (*H*_O_), expected heterozygosity (*H*_E_), and unbiased expected heterozygosity (*uH*_E_) by GENALEX v.6.5 (Peakall and Smouse 2012). As each summary statistic was not normally distributed nor homogeneous, we carried out the Kruskal–Wallis *H* tests in SPSS 26 (SPSS Inc., Chicago, IL, USA) to test the significance of genetic diversity.

**FIGURE 3:**
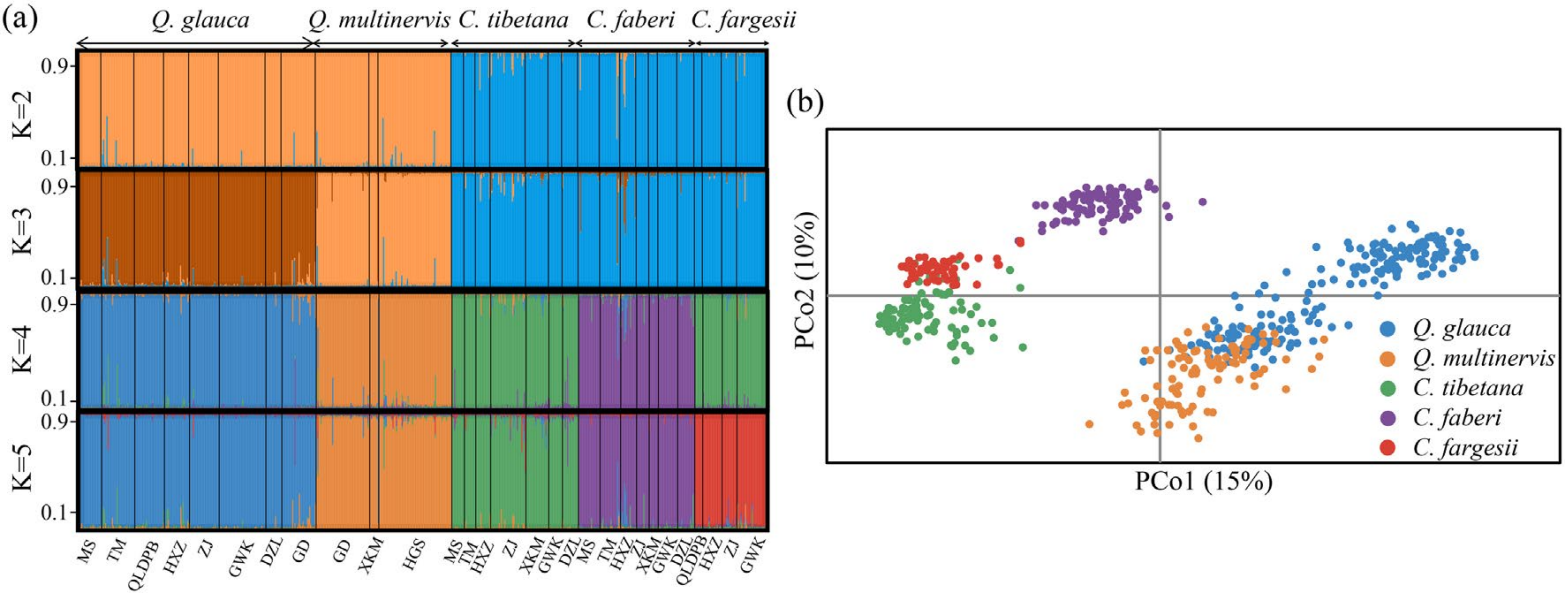
Genetic assignment and differentiation of the five Fagaceae species based on nuclear microsatellite (nSSR) data. (a) Histogram assignments of all individuals, each bar represents a single individual, with portions of the bar colored depending on the ancestry proportions estimated. Each species is indicated on the top and population codes below the histogram. (b) Principal coordinates analysis (PCoA) for pure individuals based on nSSR data.

We performed Bayesian cluster analysis inference of the population structure for all individuals of five Fagaceae species using STRUCTURE v.2.3 (Pritchard et al. 2000). We performed 20 independent runs for each value of *K* (1–10) using 200,000 generations for the Markov Chain Monte Carlo cycles (MCMC) and 100,000 generations for the burn‐in cycles. We estimated the most likely number of clusters (*K*) using Δ*K* and LnP (*K*) statistics in the STRUCTURE HARVESTER (Earl and Vonholdt 2012; Evanno et al. 2005). In order to further explore possible genetic clusters, we provided STRUCTURE plots for different *K* values for visual comparison using DISTRUCT (Figure 3a) (Rosenberg 2004). We used admixture coefficient (*Q*) values to determine whether individuals were purebred or hybrid. We selected the threshold *Q* values of 0.9/0.1 as suggested by other oak studies (Lepais et al. 2009; Peñaloza‐Ramírez et al. 2010; Qi et al. 2024). Individuals with *Q* values more than 0.9 or smaller than 0.1 were classified as purebreds, while those with *Q* values between 0.1 and 0.9 were considered intermediate or hybrids. The analyses were conducted based on pure and all individuals by genetic assignment in this study respectively to evaluate the potential influence of hybridization on genetic diversity and differentiation, fine‐scale SGS, and leaf shape variation. We also performed principal coordinate analysis (PCoA) of the genetic distance matrix and plotted the first two eigenvectors to visualize genetic proximities of individuals using GENALEX v.6.5 (Peakall and Smouse 2012).

We conducted hierarchical analysis of molecular variance (AMOVA) to quantify the degree of genetic differentiation between species and among populations using ARLEQUIN v.3.5 (Excoffier and Lischer 2010). Subsequently, we evaluated the significance of genetic differentiation using 10,000 permutations in ARLEQUIN v.3.5.

#### 2.4.2 Inference of Fine‐Scale Spatial Genetic Structure (Fine‐Scale SGS)

We assessed the fine‐scale SGS through individual‐level spatial autocorrelation analysis using SPAGeDi v.1.5 (Hardy and Vekemans 2002). We first divided the spatial distances of each species into 10 distance classes to ensure an equal number of pairwise comparisons for each class. We then estimated the average of the pairwise kinship coefficient (*F*_ij_) for each distance class using Nason's estimator, which represents the genetic relatedness between individuals i and j (Loiselle et al. 1995). The significance of *F*_ij_ at each distance class was tested by a 95% confidence interval derived from 10,000 permutations. Next, we quantified the intensity of fine‐scale SGS using the *S*_p_ statistic to enable direct comparison of fine‐scale SGS (Vekemans and Hardy 2004). The *S*_p_ statistic is calculated as *S*_p_ = −*b*/(1 − *F*_1_), where *b* is the slope of the regression of *F*_ij_ values on the natural logarithm of the spatial distance among individuals, and *F*_1_ is the average of *F*_ij_ in the first distance class (Vekemans and Hardy 2004). Finally, we visualized fine‐scale SGS by plotting the relationship between *F*_ij_ and geographical distance.

#### 2.4.3 Leaf Morphological Analysis

For traditional morphometric methods, we first carried out the Shapiro–Wilk test and Levene test (package car, Fox and Weisberg 2019) to test for normality and homogeneity of variance respectively. We then conducted a one‐way analysis of variance (one‐way ANOVA) to test the differences among species for the seven traditional leaf morphological traits (Li et al. 2022). Finally, we calculated the mean and standard deviation (SD) using SPSS 26. We also estimated the coefficient of variation (CV) to compare and quantify the degree of leaf shape variation for each trait among species. Because LA is not always independent of interspecies differences in LM, we used subsequent statistical analyses as log_10_‐transformed data (Niklas et al. 2007). We conducted preliminary regression analyses to calculate the standardized major axis slopes and intercepts (*α* and log_*β*_, respectively) for log–log linear relations between LA and LM for each species.

For GMMs analysis, we first conducted generalized procrustes analysis (GPA) to extract leaf shape and leaf size by minimizing the sum of squared distances among corresponding landmarks (Klingenberg et al. 2002; Rohlf and Slice 1990; Viscosi and Cardini 2011). We then removed outliers that significantly deviated from the averages. Next, we separated symmetric (the variation in averages of the original and mirrored configurations) and asymmetric components (the differences between original and mirrored configurations) for the leaf shape data (Klingenberg et al. 2002; Mardia et al. 2000). We created a wireframe for visualizing leaf shape changes. Finally, we created covariance matrices at the tree level for subsequent multivariate statistical analysis. We performed principal component analysis (PCA) on symmetric and asymmetric components to identify the leaf shape variations among species (Klingenberg 2011; Klingenberg et al. 2012). We conducted two‐block partial least squares (2B‐PLS) analysis on symmetric and asymmetric components to assess allometric patterns of covariation between leaf size and shape (Rohlf and Corti 2000). We performed canonical variate analysis (CVA) to detect differences among species using Mahalanobis distances for permutation tests (*T* ^2^ statistics; 10,000 permutations per test) (Viscosi and Cardini 2011). We performed discriminant analysis (DA) to distinguish the species using cross‐validated scores classification tables with *T* ^2^ statistics (*p* value for tests with 1000 permutations < 0.0001) (Klingenberg 2011).

## 3 Results

### 3.1 Genetic Diversity and Differentiation

The observed heterozygosity (*H*_O_) ranged from 0.54 to 0.60, and the expected heterozygosity (*H*_E_) ranged from 0.54 to 0.62 (Tables S3 and S4). Bayesian clustering indicated that *K* equals three as the optimal number of clusters (Figure S1), grouping all individuals into three clusters: one corresponded to *Q. glauca* , one to *Q. multinervis*, and the other to the three species of *Castanopsis* (Figure 3a). When *K* equals five, each species was classified into a distinct cluster (Figure 3a). Based on a threshold *Q* value of 0.9/0.1, 79 individuals were identified as admixture: *Q. glauca* × *multinervis* (13), *Q. glauca* × *C. tibetana* (12), *Q. glauca* × *C. faberi* (7), *Q. glauca* × *C. fargesii* (4), *Q. multinervis* × *C. tibetana* (8), *Q. multinervis* × *C. faberi* (3), *Q. multinervis* × *C. fargesii* (5), *C. tibetana* × *faberi* (13), *C. tibetana* × *fargesii* (7), *C. faberi* × *fargesii* (7) (Figure 3a). The PCoA analysis was consistent with the STRUCTURE analysis, showing five clusters corresponding to the five Fagaceae species (Figure 3b; Figure S2).

The results of AMOVA indicated a high level of genetic differentiation among species (Table 1; Table S5). The intraspecies analysis revealed that *Q. glauca* exhibited the highest genetic differentiation, while *C. fargesii* showed the lowest genetic differentiation (Table 1; Table S5). The majority of the genetic variation occurred within populations (Table 1; Table S5). The results of genetic diversity and differentiation for all individuals were similar to those for pure individuals.

**TABLE 1:** Analysis of molecular variance (AMOVA) for pure individuals of the five Fagaceae species based on nuclear microsatellite (nSSR) data.

Source of variation	df	SS	VC	V%	*F*_ST_
*Q. glauca*					
Among populations	7	138.99	0.37	12.26	0.12
Within populations	366	976.97	2.67	87.74	
*Q. multinervis*					
Among populations	2	37.05	0.25	7.42	0.07
Within populations	213	677.08	3.18	92.58	
*C. tibetana*					
Among populations	6	35.06	0.14	5.29	0.05
Within populations	167	411.65	2.46	94.71	
*C. faberi*					
Among populations	6	27.16	0.09	3.69	0.04
Within populations	165	387.71	2.35	96.31	
*C. fargesii*					
Among populations	3	9.96	0.04	1.43	0.01
Within populations	104	251.74	2.42	98.57	
All species					
Among species	4	856.85	1.00	24.12	
Among populations within species	24	279.52	0.26	6.23	0.30
Within populations	1015	2936.17	2.89	69.65	

*Note:* Significance tests (1000 permutations) showed all fixation indices were significant (*p* < 0.001).

Abbreviations: df, degrees of freedom; *F*_ST_, differentiation among populations; SS, sum of squares; V%, percentage of variation; VC, variance component.

### 3.2 Fine‐Scale Spatial Genetic Structure (Fine‐Scale SGS)

**FIGURE 4:**
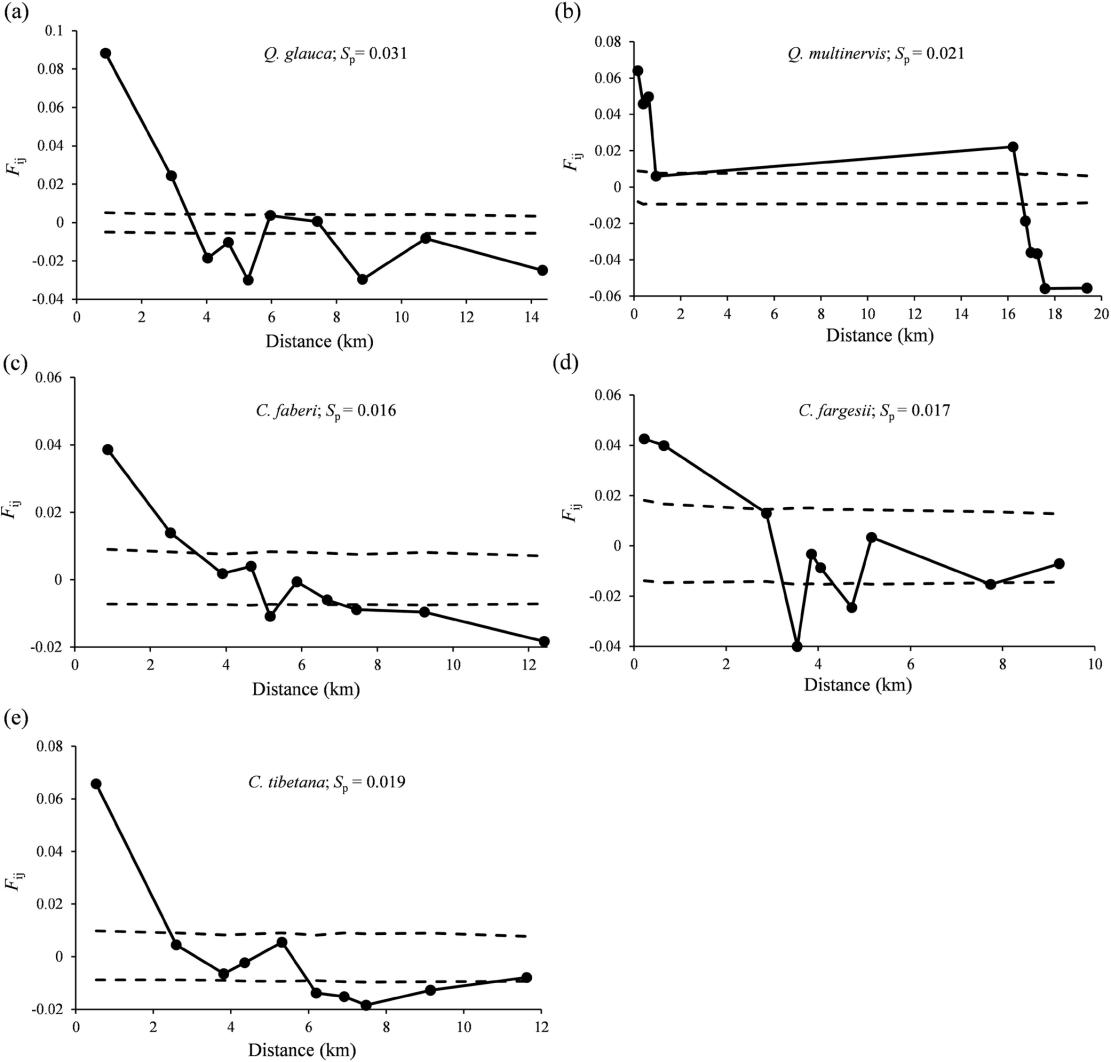
Fine‐scale spatial genetic structure (fine‐scale SGS) for pure individuals of the five Fagaceae species based on nuclear microsatellite (nSSR) data. The pairwise kinship coefficient (*F*_ij_) was plotted against geographical distances (km). The dotted lines indicate the 95% confidence interval for the pairwise kinship coefficient (*F*_ij_) values (shown by solid lines). *S*_p_ statistic represents the intensity of fine‐scale SGS for each species.

We detected significant fine‐scale SGS in both *Quercus* and *Castanopsis* species (*p* < 0.001) (Figure 4; Figure S3). For the *Quercus* species, *Q. glauca* presented a stronger fine‐scale SGS with significant *F*_ij_ extending up to 2.91 km, while *Q. multinervis* displayed significant *F*_ij_ up to 0.63 km. Among the *Castanopsis* species, *C. faberi* exhibited significant *F*_ij_ up to 2.53 km, *C. fargesii* up to 0.65 km and *C. tibetana* up to 0.53 km. For all species, *F*_ij_ peaked at the first distance class and decreased with the increased distance.

*Q. glauca* had the highest *F*_1_ value at the first distance class of 0.89 km. The *S*_p_ statistic, indicating the intensity of fine‐scale SGS, was highest in *Q. glauca* (*S*_p_ = 0.031) and lowest in *C. faberi* (*S*_p_ = 0.016). The fine‐scale SGS results for all individuals were similar to those for pure individuals.

### 3.3 Leaf Shape Variation

One‐way ANOVA showed significant differences in the seven traditional leaf morphological traits among species (Tables S6 and S7). The seven traditional leaf morphological traits showed that the CV ranged from 17.80% to 96.81%, LM and LA had higher variability than other traits (Table 2; Table S8). *C. tibetana* had the largest leaves, while *C. fargesii* had the smallest leaves (Table 2; Table S8).

**TABLE 2:** Means and standard deviations (SD) of seven traditional leaf morphological traits for pure individuals of the five Fagaceae species and coefficient of variation (CV) for each trait.

Traditional leaf morphological traits	*Q. glauca*	*Q. multinervis*	*C. tibetana*	*C. faberi*	*C. fargesii*	CV (%)
Leaf length (LL) (cm)	12.31 ± 1.85	13.45 ± 1.86	22.16 ± 3.71	11.33 ± 1.72	10.17 ± 1.58	32.24
Petiole length (PL) (cm)	2.14 ± 0.50	1.74 ± 0.40	2.15 ± 0.43	0.78 ± 0.28	0.78 ± 0.20	41.62
Leaf width (LW) (cm)	2.47 ± 0.45	2.42 ± 0.39	4.41 ± 0.77	1.93 ± 0.30	1.58 ± 0.24	37.72
Length of lamina from base to widest point (WP) (cm)	6.10 ± 1.19	6.46 ± 1.21	11.43 ± 2.64	4.88 ± 1.06	4.68 ± 0.79	39.60
Leaf mass (LM) (g)	0.68 ± 0.24	0.62 ± 0.19	2.59 ± 0.96	0.43 ± 0.14	0.30 ± 0.09	96.81
Leaf area (LA) (cm^2^)	41.00 ± 12.33	43.61 ± 11.63	135.07 ± 42.88	30.09 ± 8.44	22.33 ± 6.05	78.89
Specific leaf area (SLA) (cm^2^·g^−1^)	61.61 ± 8.28	72.14 ± 10.45	53.54 ± 8.00	70.80 ± 9.36	75.28 ± 9.96	17.80

Statistically significant scaling relationships between LA and LM were observed. LA and LM were correlated, with LA generally scaling less than a one‐to‐one ratio with increasing LM (Table 3; Table S9). *C. fargesii* had the highest scaling exponents, while the scaling exponents of other species were similar (Table 3; Table S9).

**TABLE 3:** Statistical parameters of the log–log linear relations between leaf area (LA) and leaf mass (LM) for pure individuals of the five Fagaceae species.

	*Q. glauca*	*Q. multinervis*	*C. tibetana*	*C. faberi*	*C. fargesii*
*α*	0.80	0.80	0.80	0.81	0.83
log_*β*_	1.75	1.80	1.80	1.77	1.78
95% CI	(0.78, 0.82)	(0.78, 0.83)	(0.76, 0.83)	(0.77, 0.85)	(0.78, 0.88)
*R* ^2^	0.84	0.80	0.84	0.79	0.81
*p*	< 0.001	< 0.001	< 0.001	< 0.001	< 0.001

*Note:* *p* value indicates statistical significance.

Abbreviations: *α*, the slope of the log_10_‐transformed LA vs. LM regression curve; *β*, the elevation of the log_10_‐transformed LA vs. LM regression curve; CI, confidence intervals; *R* ^2^, coefficient of determination.

**FIGURE 5:**
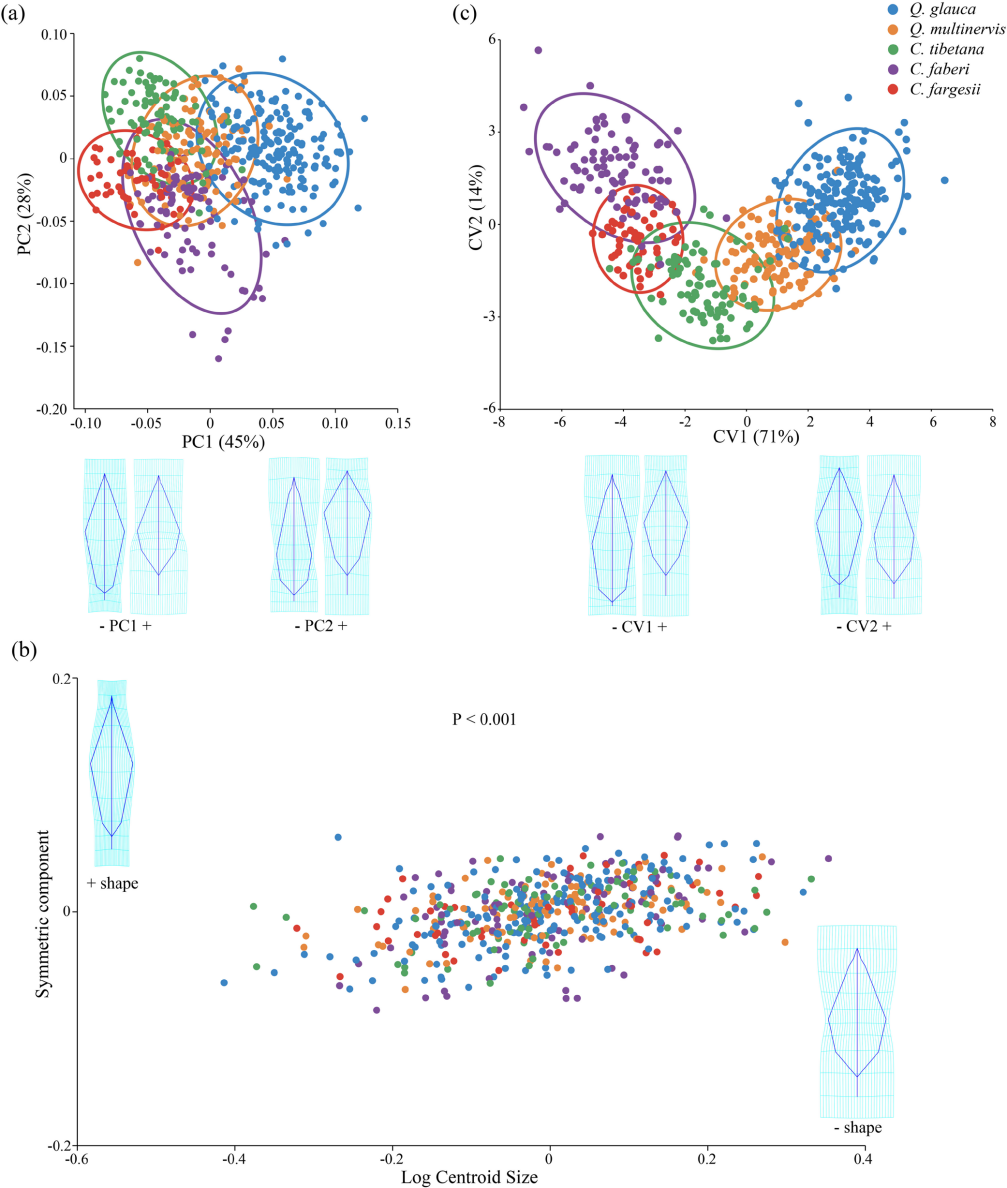
Leaf geometric morphometric analysis at the tree level for pure individuals of the five Fagaceae species. (a) Scatter plot of principal component analysis (PCA) for symmetric components with 90% confidence ellipses. Transformation grid below shows extreme leaf shapes along PCs. (b) Scatter plot of two‐block partial least squares (2B‐PLS) analysis for symmetric components. Transformation grid shows leaf shapes along PLS axis. (c) Scatter plot of canonical variate analysis (CVA) with 90% confidence ellipses. Transformation grid below shows extreme leaf shapes along CVs.

For GMMs analysis, the results of PCA for symmetric components indicated partial separation of *Q. glauca* , *C. tibetana* , and *C. faberi*, with PC1 accounted for 45% and PC2 accounted for 28% (Figure 5a; Figure S4a). The leaf shape variations were associated with the relative length of the petiole, the upper part of the leaf, and the leaf base and apex (Figure 5a; Figure S4a). PCA for asymmetric components indicated fully and densely overlap among species, and the leaf shape variation showed no regular pattern (Figures S5a and S6a). 2B‐PLS analysis showed significant allometric patterns in the symmetric components (Figure 5b; Figure S4b), while not in the asymmetric components (Figures S5b and S6b). As leaf size increased, the relative length of the petiole decreased, the leaf shape changed from subelliptical to lanceolate, and overall narrowed (Figure 5b; Figure S4b). CVA indicated the five species formed five groups along the CV axis, with the first two CVs accounted for 85% of the total leaf shape variation (Figure 5c; Figure S4c). CVA showed that *Q. glauca* had relatively longer petioles and narrower leaf base compared to *C. faberi* (Figure 5c; Figure S4c). DA showed high accuracy in leaf species identification, ranging from 93% to 100%, with the lowest discrimination rate between *Q. glauca* and *Q. multinervis* at 94.65% and 92.59% (Figures S7 and S8). The results of leaf shape variation for all individuals were similar to those for pure individuals.

## 4 Discussion

In this study, we investigated both fine‐scale SGS and leaf shape variation in five Fagaceae species from the genera *Quercus* and *Castanopsis* in Wuyishan National Park. Our results revealed that *Castanopsis* and *Quercus* species exhibit distinct levels of genetic diversity, and that *Quercus* species exhibit stronger fine‐scale SGS than *Castanopsis* species. Leaf morphological analysis shows that *Quercus* and *Castanopsis* species differ in leaf shape; LA and LM display the most significant interspecific differences. All five species show a pattern of diminishing returns in leaf trait scaling. Species delimitation based on molecular markers was more distinct than that based on morphological traits. Given the habitat fragmentation in this region, our findings underscore the importance of conserving fine‐scale SGS and considering leaf shape variation in the design of effective conservation strategies.

### 4.1 Patterns of Fine‐Scale Spatial Genetic Structure

*Castanopsis* and *Quercus* species exhibit distinct levels of genetic diversity (Table S3). This pattern is consistent with previous studies, where the expected heterozygosity of *Castanopsis* species generally ranges from 0.69 to 0.72 (e.g., *C. acuminatissima* , Blakesley et al. 2004; *C. chinensis* , He et al. 2013), while *Quercus* species generally ranges from 0.31 to 0.54 (e.g., *Q. geminata* , Ainsworth et al. 2003; *Q*. *fabri*, Chen et al. 2021).

The intensity of fine‐scale SGS (*S*_p_) across the five species ranged from 0.016 to 0.031 (Figure 4), placing them in the upper‐medium range compared with previous studies in Fagaceae species (e.g., *Q. ilex* , *S*_p_ = 0.004; *Q. suber* , *S*_p_ = 0.023; Soto et al. 2007). The intensity of fine‐scale SGS is affected by pollen and seed dispersal abilities (Born et al. 2008) and is positively correlated with the degree of limited gene flow (Curtu et al. 2015). We found that *Quercus* species exhibit higher intensity of fine‐scale SGS than *Castanopsis* species, consistent with more limited gene flow. Studies show that *Quercus* species possess typically limited pollen dispersal distances of approximately 65 m (e.g., *Q. lobata* , Sork et al. 2002), and most seeds are dispersed within 20 m (e.g., *Q. liaotungensis*, Li and Zhang 2003). In contrast, *Castanopsis* species appear to rely more on a high potential for long‐distance dispersal of pollen (e.g., *C. sieboldii* , Nakanishi et al. 2012), and most seeds' dispersal distances might exceed 100 m (e.g., *C. chinensis* , Wang et al. 2014). These dispersal strategies have direct consequences for genetic diversity and spatial genetic patterns (Islam et al. 2014). Limited dispersal in *Quercus* species can lead to stronger local genetic clustering and increased genetic drift, potentially reducing within‐population genetic diversity over time (Montalvo et al. 1997). In contrast, greater gene flow in *Castanopsis* species may buffer against genetic erosion and promote higher heterozygosity (Wu et al. 2024). In addition, the fine‐scale SGS results remained nearly unchanged after the removal of the mixed individuals (Figure 4; Figure S3), indicating that hybridization had a negligible influence on fine‐scale SGS in this study. This finding is consistent with observations from other mixed oak forests (Curtu et al. 2015).

### 4.2 Leaf Shape Variation Associated With Ecological Adaptation

Leaf shape variation often reflects the adaptability of plant species to their environment, particularly in relation to water and light utilization (Blue and Jensen 1988; Du et al. 2022; Qi et al. 2025; Ramírez‐Valiente et al. 2017; Zhang et al. 2025). The seven traditional leaf morphological traits exhibited significant variations among five Fagaceae species (Table S6), with an average CV of 49.24% (Table 2). Compared with other oak studies (e.g., *Q. guyavifolia*, 20.99%, Sun et al. 2016; *Q*. *castaneifolia*, 29.99%, Aliyeva 2021), the five Fagaceae species showed greater variation in leaf shape. Notably, LA and LM exhibited the highest CV values, suggesting that these two traits may be more responsive to the species differentiation. Further investigation of the scaling relationships between LA and LM revealed significant diminishing returns in five Fagaceae species (Table 3), with similar diminishing returns also observed in other Fagaceae studies (e.g., *Q. glauca* , Guo et al. 2022; *Q. serrata* , Qi et al. 2025; *Q. multinervis*, Zhu et al. 2019). The concept of diminishing returns indicates a faster increase in LM than LA, with increased investment in dry mass of per unit leaf area reflecting higher investment to inert mass components (which increase leaf size but contribute minimally to photosynthetic capacity), such as cellulose, lignin, and sclerenchyma (Niklas et al. 2007). Among the five species, *C. fargesii* not only exhibited the strongest diminishing returns (Table 3), but also had the smallest leaves (i.e., smallest LL, PL, and LA) (Table 2), which may reduce transpiration water loss through a smaller surface area (Casper et al. 2001; Qin et al. 2018). This combination of strongest diminishing returns and smallest leaves may enhance the species' competitiveness in resource‐limited environments (Goud et al. 2023; Qi et al. 2025; Wright et al. 2004).

GMMs results revealed that symmetric components of leaf shape more effectively captured morphological variation across the five species than asymmetric components (Figure 5; Figure S5), a phenomenon also found in other oak studies, such as *Q. dentata* (Yang et al. 2022), *Q. cerris* and *Q. frainneto* (Jovanović et al. 2022), and *Q. aquifolioides* (Li et al. 2021). Leaf morphology between the genera *Quercus* and *Castanopsis* species showed a clear separation in symmetric components, with partial overlap (Figure 5). *Quercus* species exhibited a greater range of leaf shape variation, characterized by longer petioles and narrower leaf base compared to *Castanopsis* species (Figure 5). One plausible explanation for this difference is their potential adaptation to varying water availability (Ramírez‐Valiente et al. 2017). Some studies indicated that *Quercus* species exhibit strong adaptation to drought conditions (e.g., *Q. robur* , Nosenko et al. 2025), while *Castanopsis* species are more adapted to humid environments and tend to be less drought‐resistant (e.g., *C. hystrix* , Shen et al. 2023). Alternatively, differences in light adaptation may also contribute. Light‐demanding *Quercus* species might enhance light capture through longer petioles (e.g., *Q. velutina* , Kusi and Karsai 2019) while *Castanopsis* species (*C. faberi* and *C. fargesii* ) might be better suited to closed‐canopy environments due to their shorter petioles (e.g., *C. fargesii* , Cornelissen 1993). Moreover, in this study, the patterns of leaf shape variation based on all individuals were almost unchanged to those obtained after excluding the mixed individuals, indicating that hybridization had little influence on leaf shape variation.

Although *Quercus* and *Castanopsis* species exhibit taxonomic ambiguity in morphology, they are clearly distinct by genetic assignment (Figures 3 and 5). This pattern, together with the strong fine‐scale SGS observed in *Quercus* and *Castanopsis* species (Figure 4), suggests that leaf shape variation does not align with the neutral genetic variation. Leaf shape variation is more likely to represent ecological adaptation and responses to local environmental conditions. In this study, although the species are distributed across a relatively small spatial scale, the genetic differentiation between *Q. glauca* and *Q. multinervis* was more obvious (Figure 3), suggesting more limited gene flow between them.

### 4.3 Conservation Implications

Integrating studies of the intensity of fine‐scale SGS and leaf shape variation is crucial for developing species conservation strategies. The five species exhibited significant fine‐scale SGS, likely reflecting local habitat fragmentation (Harata et al. 2012), which might drive loss of genetic diversity (Moreira et al. 2009) and thereby reduce species' adaptability to environmental change (Du 2023). Moreover, variation in leaf traits represents phenotypic responses of plants to environmental pressures (Du et al. 2022; Qi et al. 2025; Zhang et al. 2025). High intensity of fine‐scale SGS and low genetic diversity as observed in *Q. glauca* and *Q. multinervis*, indicates limited adaptive capacity. These species also exhibit weak diminishing returns, suggesting relatively reduced investment in leaf mass per unit area under resource‐limited conditions. However, their broad range of leaf shape variation and long petioles suggest adaptive advantages in drought‐prone, high‐light environments. For these species, *ex situ* conservation strategies in drought, sufficient light environments are recommended. In contrast, *C. tibetana* , *C. faberi* and *C. fargesii* exhibit low intensity of fine‐scale SGS and high genetic diversity, indicating greater adaptive potential. These species show strong diminishing returns, which align with a resource‐conservative strategy that may be advantageous in competitive or resource‐limited environments. Their narrow leaf shape variation and short petioles suggest adaptation to humid, shaded habitats. *In situ* conservation, combined with effort to maintain higher humidity and reduce light exposure, would be appropriate for these species. Overall, our findings advocate for prioritizing genetic factors in conservation planning, while considering phenotypic traits, such as leaf shape variation, as supplementary indicators of adaptive capacity. In the near future, genome‐wide data from next generation sequencing with detailed microenvironmental information will greatly enhance our understanding of environmental adaptability and support more effective conservation efforts.

## Author Contributions

**Rongle Wang:** conceptualization (supporting), data curation (lead), formal analysis (lead), investigation (lead), methodology (equal), project administration (supporting), validation (lead), visualization (equal), writing – original draft (lead), writing – review and editing (equal). **Yanjun Luo:** conceptualization (equal), data curation (supporting), investigation (equal), methodology (equal), visualization (equal), writing – review and editing (equal). **Min Qi:** data curation (equal), formal analysis (supporting), methodology (equal), visualization (equal), writing – review and editing (equal). **Yi Zhang:** visualization (equal), writing – review and editing (equal). **Jiawen Zhang:** data curation (equal), investigation (equal), writing – review and editing (equal). **Yibo Luo:** funding acquisition (supporting), writing – review and editing (equal). **Fang K. Du:** conceptualization (lead), funding acquisition (lead), investigation (supporting), project administration (lead), resources (lead), supervision (lead), validation (lead), visualization (equal), writing – original draft (supporting), writing – review and editing (lead).

## Funding

This research was supported by the National Key Programme of Research and Development, the Ministry of Science and Technology (2022YFF1301401) to YBL, the Special Program for the Institute of National Parks, Chinese Academy of Sciences (KFJ‐STS‐ZDTP‐2022‐001), and the National Science Foundation of China (No. 42571062 and U2571202) to FD.

## Conflicts of Interest

The authors declare no conflicts of interest.

## Supporting information


**Figure S1:** Population clusters identified for all individuals of the five Fagaceae species with STRUCTURE software. (a) The functional relationship between Δ*K* and *K*. (b) The functional relationship between LnP (*K*) and *K*.
**Figure S2:** Principal coordinates analysis (PCoA) for all individuals of the five Fagaceae species based on nuclear microsatellite (nSSR) data.
**Figure S3:** Fine‐scale spatial genetic structure (fine‐scale SGS) for all individuals of the five Fagaceae species based on nuclear microsatellite (nSSR) data. The pairwise kinship coefficient (*F*_ij_) was plotted against geographical distances (km). The dotted lines indicate the 95% confidence interval for the pairwise kinship coefficient (*F*_ij_) values (shown by solid lines). *S*_p_ statistic represent the intensity of fine‐scale SGS for each species.
**Figure S4:** Leaf geometric morphometric analysis at the tree level for all individuals of the five Fagaceae species. (a) Scatter plot of principal component analysis (PCA) for symmetric components with 90% confidence ellipses. Transformation grid below shows extreme leaf shapes along PCs. (b) Scatter plot of two‐block partial least squares (2B‐PLS) analysis for symmetric components. Transformation grid shows leaf shapes along PLS axis. (c) Scatter plot of canonical variate analysis (CVA) with 90% confidence ellipses. Transformation grid below shows extreme leaf shapes along CVs.
**Figure S5:** Leaf geometric morphometric analysis at the tree level for pure individuals of the five Fagaceae species based on asymmetric components. (a) Scatter plot of principal component analysis (PCA). Transformation grid below shows extreme leaf shapes along PCs. (b) Scatter plot of two‐block partial least squares (2B‐PLS) analysis.
**Figure S6:** Leaf geometric morphometric analysis at the tree level for all individuals of the five Fagaceae species based on asymmetric components. (a) Scatter plot of principal component analysis (PCA). Transformation grid below shows extreme leaf shapes along PCs. (b) Scatter plot of two‐block partial least squares (2B‐PLS) analysis.
**Figure S7:** Discriminant analysis (DA) at the tree level of leaf shape among pairwise comparisons for pure individuals of the five Fagaceae species.
**Figure S8:** Discriminant analysis (DA) at the tree level of leaf shape among pairwise comparisons for all individuals of the five Fagaceae species.
**Table S1:** The geographic and sample information of the five Fagaceae species used in the study.
**Table S2:** Detailed information for the 63 pairs of nuclear microsatellite (nSSR) primers.
**Table S3:** Genetic diversity for pure individuals of the five Fagaceae species estimated based on nuclear microsatellite (nSSR) data.
**Table S4:** Genetic diversity for all individuals of the five Fagaceae species estimated based on nuclear microsatellite (nSSR) data.
**Table S5:** Analysis of molecular variance (AMOVA) for all individuals of the five Fagaceae species based on nuclear microsatellite (nSSR) data.
**Table S6:** One‐way analysis of variance (one‐way ANOVA) for seven traditional leaf morphological traits for pure individuals of the five Fagaceae species.
**Table S7:** One‐way analysis of variance (one‐way ANOVA) for seven traditional leaf morphological traits for all individuals of the five Fagaceae species.
**Table S8:** Means and standard deviations (SD) of seven traditional leaf morphological traits for all individuals of the five Fagaceae species and coefficient of variation (CV) for each trait.
**Table S9:** Statistical parameters of the log–log linear relations between leaf area (LA) and leaf mass (LM) for all individuals of the five Fagaceae species.

## Data Availability

Genotyping data can be found in https://doi.org/10.6084/m9.figshare.29126033, leaf traditional morphological data can be obtained in https://doi.org/10.6084/m9.figshare.29124053, and leaf geometric morphological data can be obtained in https://doi.org/10.6084/m9.figshare.29124047. Photographs of sampling sites can be obtained at https://www.oakofchina.org/photo‐of‐sampling/.
