# Supplementary material for: Fine‐Scale Spatial Genetic Structure and Leaf Shape Variation in Five Fagaceae Species: Insights Into Conservation and Adaptation
Source: Ecol Evol. 2026 Feb 18;16(2):e72863. doi: 10.1002/ece3.72863 (PMC12914225; doi:10.1002/ece3.72863)
Supplement: Supplementary file 1 — Figure S1: Population clusters identified for all individuals of the five Fagaceae species with STRUCTURE software. (a) The functional relationship between ΔK and K. (b) The functional relationship between LnP (K) and K. Figure S2: Principal coordinates analysis (PCoA) for all individuals of the five Fagaceae species based on nuclear microsatellite (nSSR) data. Figure S3: Fine‐scale spatial genetic structure (fine‐scale SGS) for all individuals of the five Fagaceae species based on nuclear microsatellite (nSSR) data. The pairwise kinship coefficient (F ij) was plotted against geographical distances (km). The dotted lines indicate the 95% confidence interval for the pairwise kinship coefficient (F ij) values (shown by solid lines). S p statistic represent the intensity of fine‐scale SGS for each species. Figure S4: Leaf geometric morphometric analysis at the tree level for all individuals of the five Fagaceae species. (a) Scatter plot of principal component analysis (PCA) for symmetric components with 90% confidence ellipses. Transformation grid below shows extreme leaf shapes along PCs. (b) Scatter plot of two‐block partial least squares (2B‐PLS) analysis for symmetric components. Transformation grid shows leaf shapes along PLS axis. (c) Scatter plot of canonical variate analysis (CVA) with 90% confidence ellipses. Transformation grid below shows extreme leaf shapes along CVs. Figure S5: Leaf geometric morphometric analysis at the tree level for pure individuals of the five Fagaceae species based on asymmetric components. (a) Scatter plot of principal component analysis (PCA). Transformation grid below shows extreme leaf shapes along PCs. (b) Scatter plot of two‐block partial least squares (2B‐PLS) analysis. Figure S6: Leaf geometric morphometric analysis at the tree level for all individuals of the five Fagaceae species based on asymmetric components. (a) Scatter plot of principal component analysis (PCA). Transformation grid below shows extreme leaf shapes along P [file ECE3-16-e72863-s001.docx]

**SUPPORTING INFORMATION**

**Figure S1.** Population clusters identified for all individuals of the five Fagaceae species with STRUCTURE software. (a) The functional relationship between ΔK and K. (b) The functional relationship between LnP (K) and K.

**Figure S2.** Principal coordinates analysis (PCoA) for all individuals of the five Fagaceae species based on nuclear microsatellite (nSSR) data.

**Figure S3.** Fine-scale spatial genetic structure (fine-scale SGS) for all individuals of the five Fagaceae species based on nuclear microsatellite (nSSR) data. The pairwise kinship coefficient (*F*_ij_) was plotted against geographical distances (km). The dotted lines indicate the 95% confidence interval for the pairwise kinship coefficient (*F*_ij_) values (shown by solid lines). *S*_p_ statistic represent the intensity of fine-scale SGS for each species.

**Figure** **S4.** Leaf geometric morphometric analysis at the tree level for all individuals of the five Fagaceae species. (a) Scatter plot of principal component analysis (PCA) for symmetric components with 90% confidence ellipses. Transformation grid below shows extreme leaf shapes along PCs. (b) Scatter plot of two-block partial least squares (2B-PLS) analysis for symmetric components. Transformation grid shows leaf shapes along PLS axis. (c) Scatter plot of canonical variate analysis (CVA) with 90% confidence ellipses. Transformation grid below shows extreme leaf shapes along CVs.

**Figure S5.** Leaf geometric morphometric analysis at the tree level for pure individuals of the five Fagaceae species based on asymmetric components. (a) Scatter plot of principal component analysis (PCA). Transformation grid below shows extreme leaf shapes along PCs. (b) Scatter plot of two-block partial least squares (2B-PLS) analysis.

**Figure S6.** Leaf geometric morphometric analysis at the tree level for all individuals of the five Fagaceae species based on asymmetric components. (a) Scatter plot of principal component analysis (PCA). Transformation grid below shows extreme leaf shapes along PCs. (b) Scatter plot of two-block partial least squares (2B-PLS) analysis.

**Figure S7.** Discriminant analysis (DA) at the tree level of leaf shape among pairwise comparisons for pure individuals of the five Fagaceae species.

**Figure** **S8.** Discriminant analysis (DA) at the tree level of leaf shape among pairwise comparisons for all individuals of the five Fagaceae species.

**Figure S1**


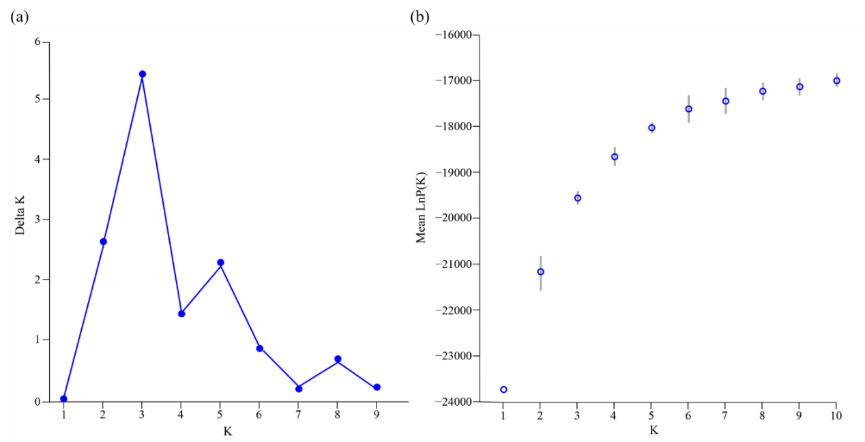


**Figure S2**

**
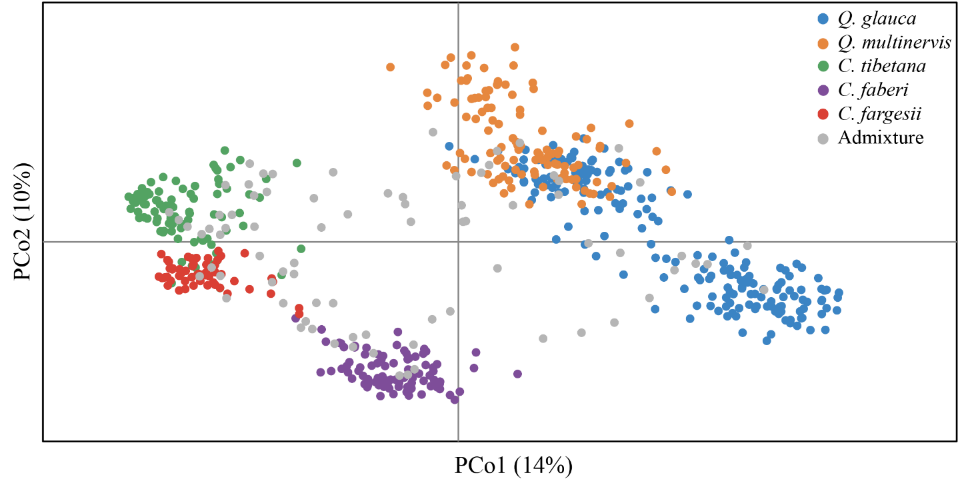
**

**Figure S3**

**
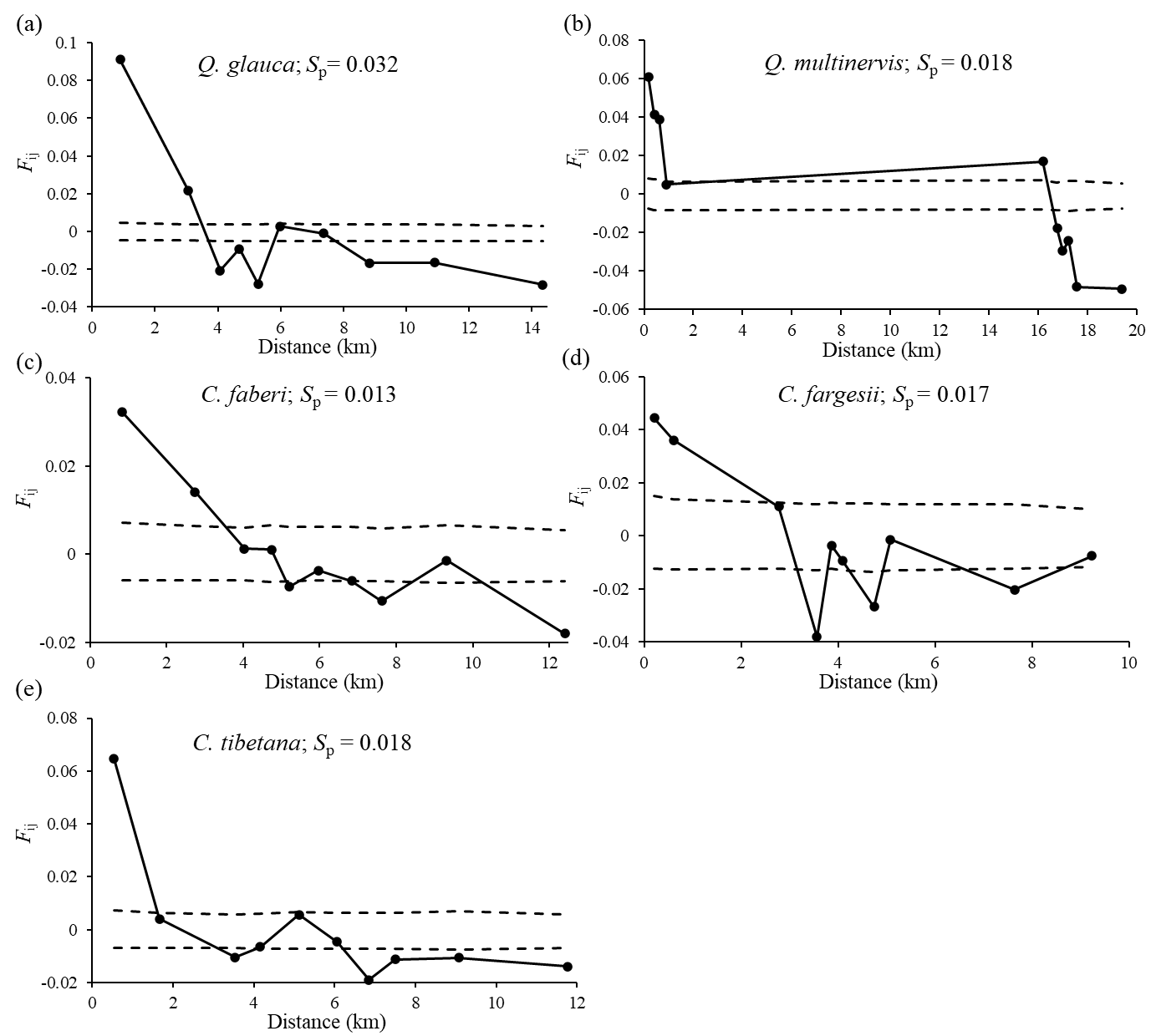
**

**Figure S4
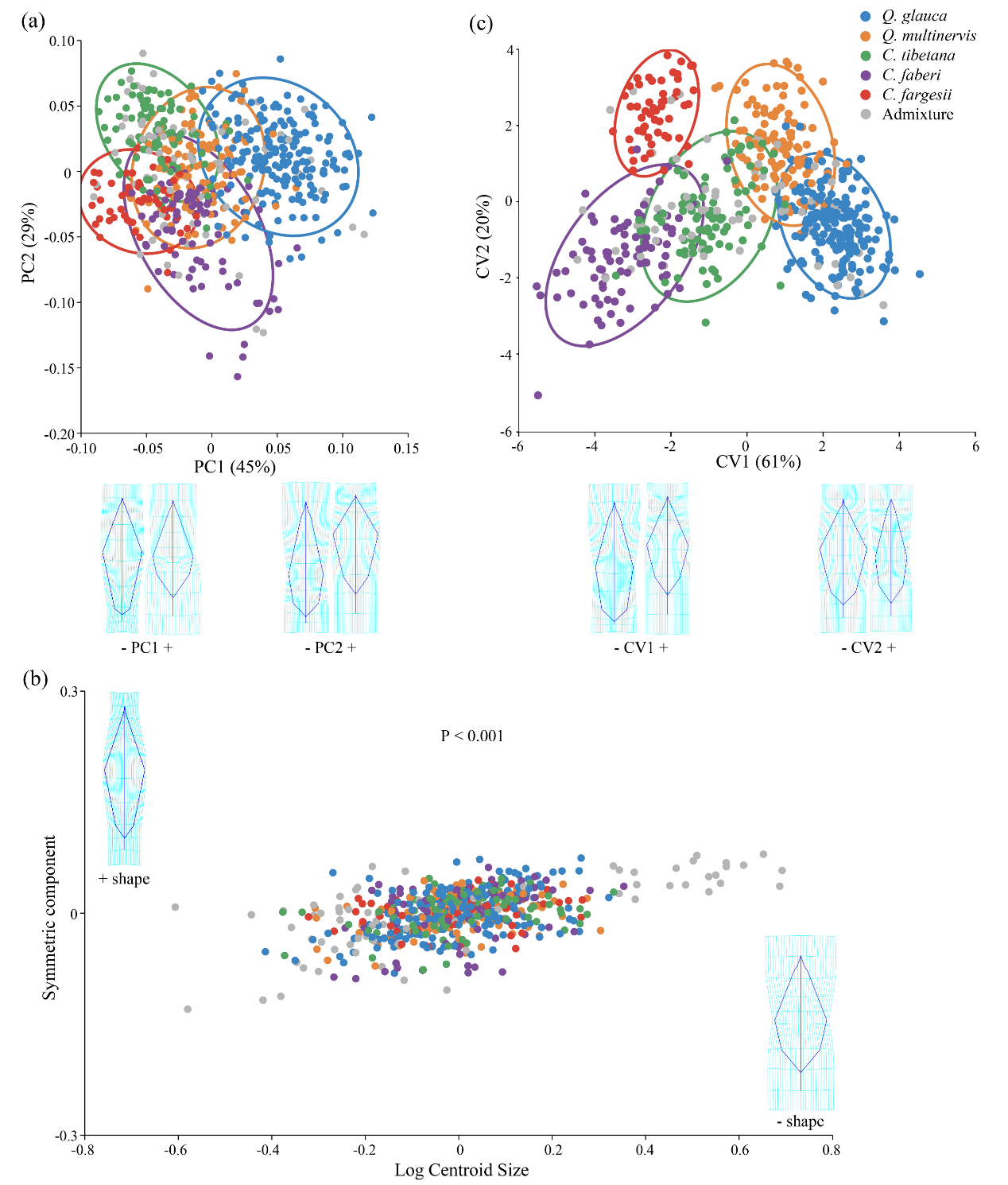
**

**Figure S5**

**
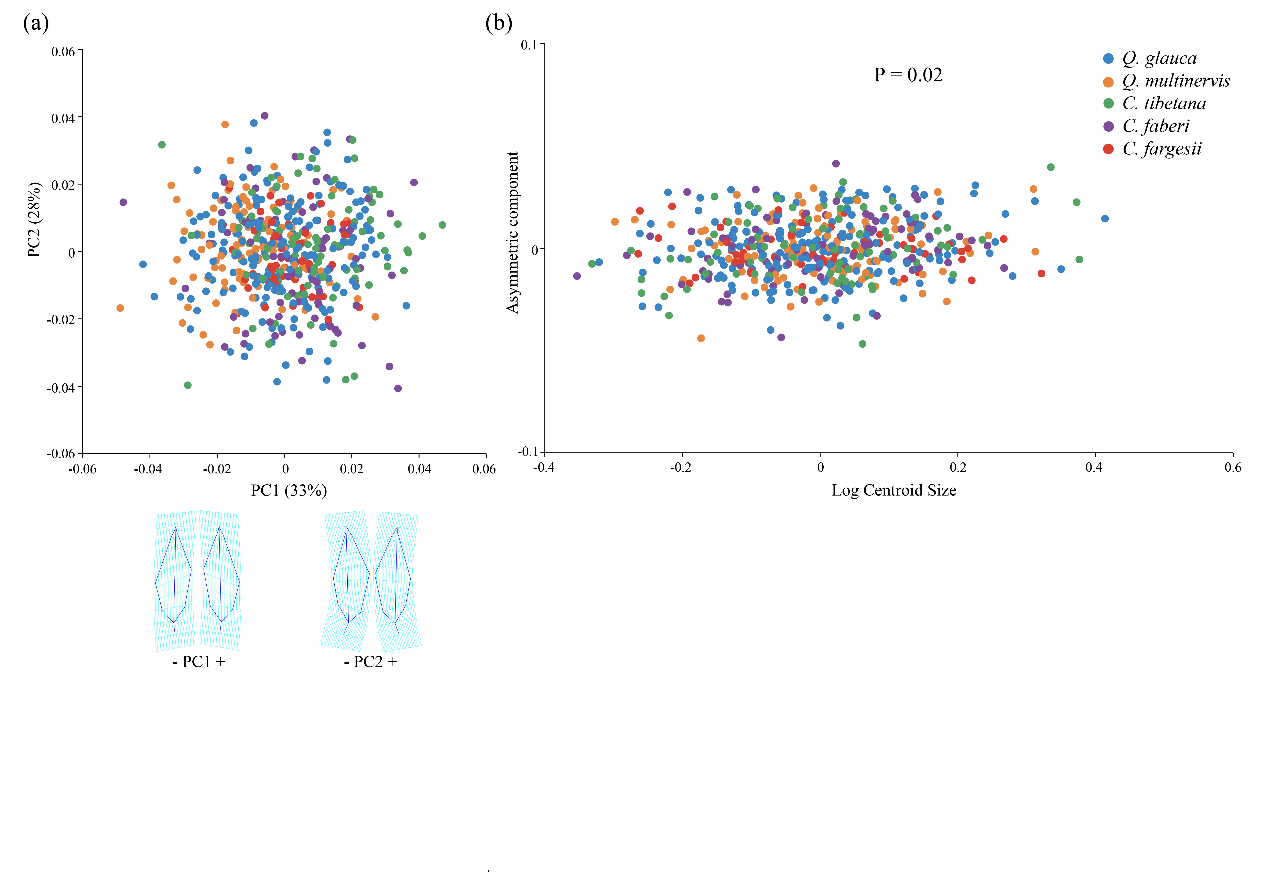
**

**Figure S6**

**
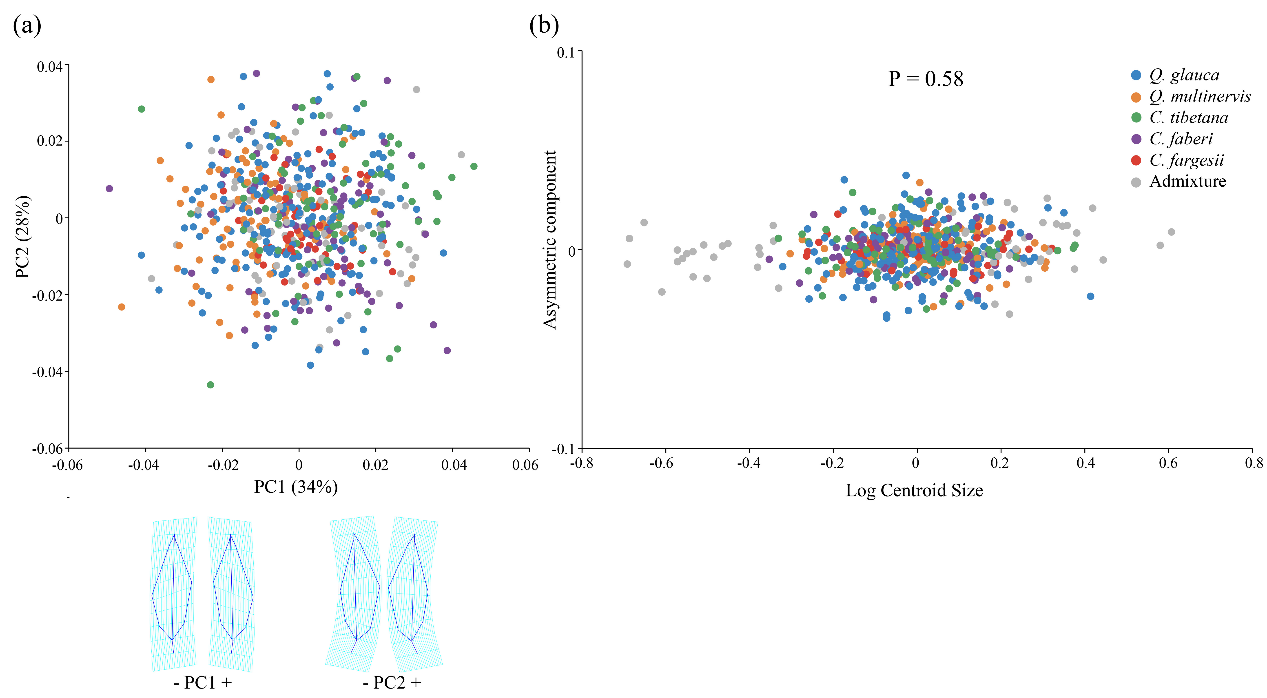
**

**Figure S7**

**
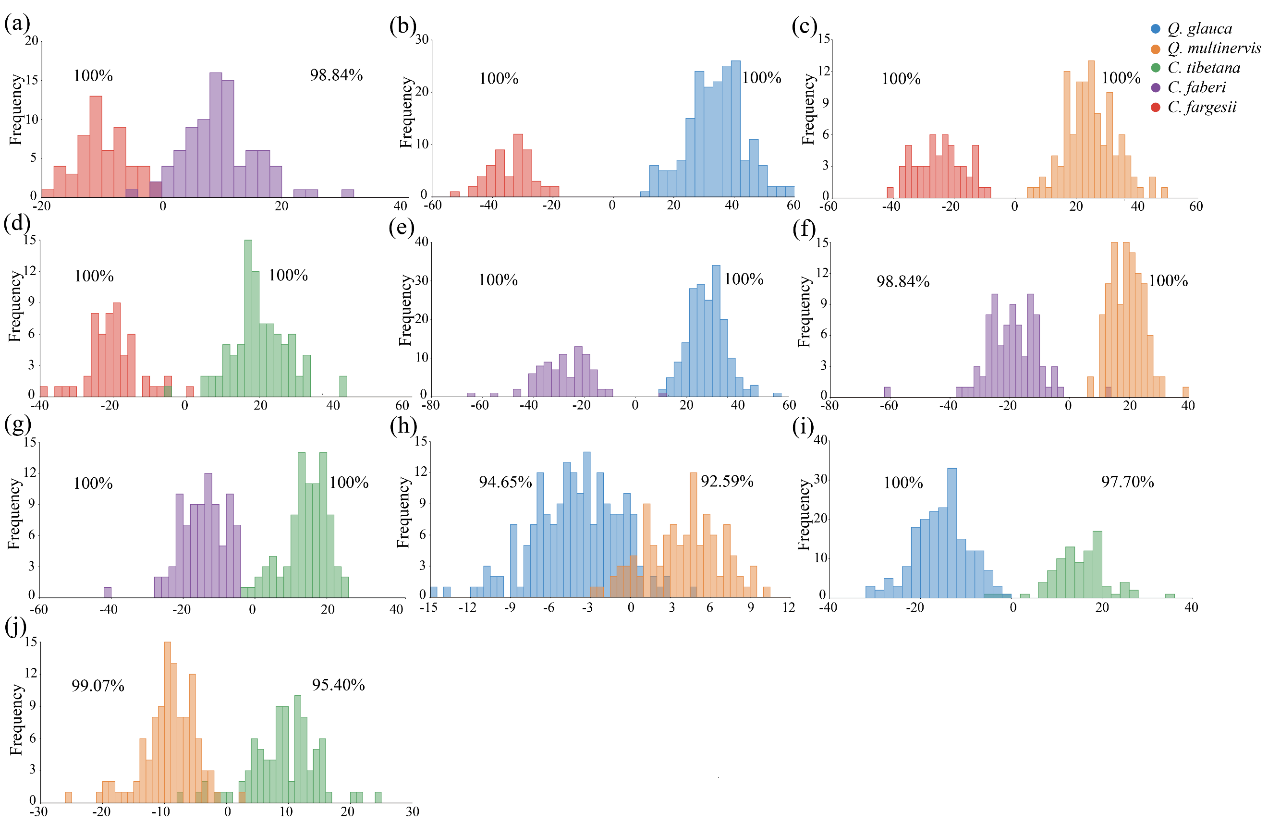
**

**Figure S8**


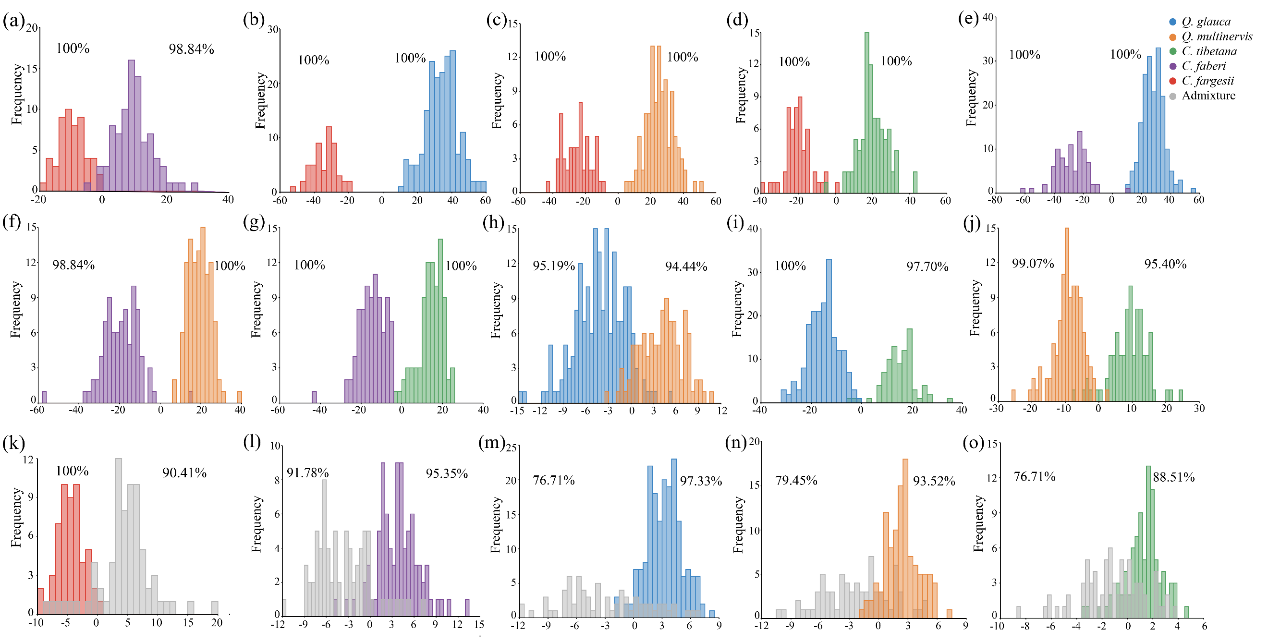


**Table S1** The geographic and sample information of the five Fagaceae species used in the study.

| Species | Code | Population | N | Longitude (E) | Latitude (N) | Altitude (m) |
| --- | --- | --- | --- | --- | --- | --- |
| *Q. glauca* | MS | Masu | 19 | 117.73 | 27.75 | 922-1284 |
|  | TM | Tongmu | 29 | 117.68 | 27.75 | 728-1268 |
|  | QLDPB | Qinglongdapubu | 26 | 117.77 | 27.67 | 309-480 |
|  | HXZ | Huangxizhou | 22 | 117.75 | 27.71 | 614-727 |
|  | ZJ | Zhangjia | 26 | 117.73 | 27.74 | 835-1000 |
|  | GWK | Guwangkeng | 41 | 117.69 | 27.71 | 533-878 |
|  | DZL | Dazhulan | 14 | 117.64 | 27.70 | 887-960 |
|  | GD | Guadun | 30 | 117.66 | 27.73 | 842-1051 |
| *Q. multinervis* | GD | Guadun | 47 | 117.63 | 27.74 | 1224-1436 |
|  | XKM | Xikengmiao | 8 | 117.68 | 27.69 | 1017-1268 |
|  | HGS | Huanggangshan | 64 | 117.76 | 27.84 | 1711-1881 |
| *C. tibetana* | MS | Masu | 11 | 117.73 | 27.74 | 926-1106 |
|  | TM | Tongmu | 10 | 117.67 | 27.76 | 805-1048 |
|  | HXZ | Huangxizhou | 13 | 117.75 | 27.71 | 604-722 |
|  | ZJ | Zhangjia | 31 | 117.72 | 27.74 | 718-1056 |
|  | XKM | Xikengmiao | 20 | 117.68 | 27.69 | 799-1275 |
|  | GWK | Guwangkeng | 12 | 117.70 | 27.71 | 533-791 |
|  | DZL | Dazhulan | 14 | 117.64 | 27.70 | 859-962 |
| *C. faberi* | MS | Masu | 19 | 117.73 | 27.75 | 1073-1403 |
|  | TM | Tongmu | 18 | 117.67 | 27.76 | 686-1058 |
|  | HXZ | Huangxizhou | 14 | 117.76 | 27.72 | 634-727 |
|  | ZJ | Zhangjia | 11 | 117.72 | 27.73 | 834-1055 |
|  | XKM | Xikengmiao | 8 | 117.69 | 27.68 | 804-1225 |
|  | GWK | Guwangkeng | 17 | 117.71 | 27.71 | 646-839 |
|  | DZL | Dazhulan | 15 | 117.64 | 27.70 | 863-960 |
| *C. fargesii* | QLDPB | Qinglongdapubu | 7 | 117.77 | 27.67 | 309-404 |
|  | HXZ | Huangxizhou | 17 | 117.76 | 27.72 | 619-727 |
|  | ZJ | Zhangjia | 13 | 117.72 | 27.74 | 841-952 |
|  | GWK | Guwangkeng | 25 | 117.71 | 27.71 | 533-841 |
| Total |  |  | 601 |  |  |  |

Note: Code, sampling population name; N, sample size.

**Table S2** Detailed information for the 63 pairs of nuclear microsatellite (nSSR) primers.

| Locus | Primer sequences (5′-3′) | Repeat motif | Size range (bp) | *T*a  (°C) | Successfully amplification | Reference |
| --- | --- | --- | --- | --- | --- | --- |
| CS43 | CTCTATCTCGCAAGCGTGTGA CCCCATTTTGTGGTTCTAAGGT | CT | 92-106 | 61 | No | Shi *et al*. 2011 |
| CS561 | ATTCATCTGGTGGGGTTT GGAGTGGAGTTAGAAGACGAT | CT | 316-352 | 55 | No | Shi *et al*. 2011 |
| CS620 | AGGTGTAGAAGGAAAAAAGC AGACTGACTCAAAACAATAAAG | CT | 118-154 | 62 | No | Shi *et al*. 2011 |
| CS627 | CGCTACTGTAACTGGAATGG CTCAATGTTTGTGTTGGTGT | CT | 190-232 | 62 | No | Shi *et al*. 2011 |
| CS687 | GTGTAAAGTTTGACCCATTG AGCAGCCACCATTTCAGTTC | CT | 129-151 | 59 | No | Shi *et al*. 2011 |
| CS721 | AAGCAATGAGTAGAGATAATGATG GAGAGATGTAGAGAATAGATGGAA | CT | 119-139 | 52 | No | Shi *et al*. 2011 |
| Cch13 | CAAACGCACCCTAAGAGGAG GTAGGTGGGCAGCGAAATAA | TC | 297-363 | 66 | No | Huang *et al*. 2009 |
| Cch15 | CCCATAACGTCTGACCCCTA CCAAAAGGGCTTCATAACCA | CT | 229-243 | 60 | No | Huang *et al*. 2009 |
| Ccu62F15 | TTGCATCCTCAGCTTTCTCA GCCCTCTCCTAACACCAATAATAC | TC | 132-137 | 50 | No | Ueno *et al*. 2003 |
| Ccu102F36 | ATCCCTACAGGCCAACCAATAA TACCGCATCAATTCTGACATCTG | TC | 342-386 | 64 | No | Ueno *et al*. 2003 |
| Ccu87F23 | CGGAACCTAACACCACTACC CTCCCTCCCTATCATACTTTGA | TC | 261-307 | 64 | No | Ueno *et al*. 2003 |
| Ccu90T17 | TACTAACCACAGCCCTAAACTGC AACCCAACGCCTCTTATGC | TC | 134-144 | 64 | No | Ueno *et al*. 2003 |
| Ccu93H17 | GGCAACATCAAGGTATTTC CTAAGCAATTTATATTGTCCAAA | TC | 171-205 | 58 | No | Ueno *et al*. 2003 |
| CsCAT3 | CACTATTTTATCATGGACGG CGAATTGAGAGTTCATACTC | AG | 224 | 50 | No | Marinoni *et al*. 2003 |
| CsCAT14 | CGAGGTTGTTGTTCATCATTAC GATCTCAAGTCAAAAGGTGTC | CA | 164 | 58 | No | Marinoni *et al*. 2003 |
| CsCAT15 | TTCTGCGACCTCGAAACCGA GCTAGGGTTTTCATTTCTAG | TC | 136 | 50 | No | Marinoni *et al*. 2003 |
| CsCAT34 | TGAGCAAGGATGGATGATGAG GGTGGTCATCATGACTGCATC | GT | 187 | 50 | No | Marinoni *et al*. 2003 |
| CsCAT41 | AAGTCAGCAACACCATATGC CCCACTGTTCATGAGTTTCT | AG | 224 | 50 | No | Marinoni *et al*. 2003 |
| Cmcs1 | ATTCATTTCCTTTGCATTGA TTTACTTGTTACTAATAGGGTCTAGC | AT | 109 | 55 | No | Sebastiani *et al*.2004 |
| Cmcs2 | GAGCCATTCCCTTTTAGAAT TTGAAAACCGGTATAGTTCG | AT | 151 | 55 | Yes | Sebastiani *et al*. 2004 |
| Cmcs3 | AGAGTAAGGTTTTATTAGTATAGA CTCGATAGTATTTGTCGAT | AT | 189 | 52 | No | Sebastiani *et al*. 2004 |
| Cmcs4 | ATTCATTCCCCTTCTATATC CCTAGTATCCCACCAATTA | TC | 110 | 55 | No | Sebastiani *et al*. 2004 |
| Cmcs14 | GGATTGTAACAAATTTTTCAGG GTGCAAGGAATGTCGAACTA | AT | 178 | 55 | No | Sebastiani et al., 2004 |
| CT110 | TTCTTCAGTTAGCCACATCG CGCTAAGTCCATACATACAACAG | TGT | 169-204 | 56 | No | Waikham *et al*.2018 |
| CT132 | TGACCCGAGCATGGTTTAT GGACGTTAGGCCTGTACATT | AAG | 126-174 | 56 | No | Waikham *et al*. 2018 |
| CT161 | AACGATACTAGCGACCTTGA GAGAAAAACGCTCTCCAAC | CAC | 136-166 | 56 | No | Waikham *et al*. 2018 |
| CG35 | GCCCAAAATTGTAATC AACCTCATGGAAACCT | AG | 122-136 | 52 | No | Tong *et al*. 2012 |
| CG37 | CAAATCTGTAGGAGGGAGC CCATGAGGCATTAGAAGGT | AG | 153-173 | 59 | No | Tong *et al*. 2012 |
| CG105 | GGTTGTTTACCATTACCCTTTC AACTCTATACACTGTTGCGTCC | CT | 161-177 | 58 | No | Tong *et al*. 2012 |
| CG128 | TTTCTCAGTCTCCGTCT GATTTGTCCCAATGTCC | AG | 163-171 | 55 | No | Tong *et al*. 2012 |
| CG199 | GAGAAGCGATGAAAGACAG AAAGATTAGGGTTCGTGAG | GA | 157-179 | 55 | No | Tong *et al*. 2012 |
| CG252 | AAGTTTACAGTCGGGTGG ATGGGATGCTAAGTGTATTG | TC | 112-158 | 55 | No | Tong *et al*. 2012 |
| CG258 | CCACCAAACCCATAG CTCTGCTCCTTACCC | AG | 135-137 | 56 | No | Tong *et al*. 2012 |
| CG371 | GAGTCACATCAACGAAT AGGGGACTAAAAGTACACTAC | TC | 172-214 | 56 | No | Tong *et al*.2012 |
| CG384 | GCATGTTGACAAGCATAG ATTTACATCAAGCCCCTC | GA | 124-130 | 53 | No | Tong *et al*.2012 |
| QM33GA1 | TGGGTTGACATTTCATTTCG GAAACGAATCATAAGGAGAG | GA | 103 | 48 | No | Isagi and Suhandono 1997 |
| QM51GA1 | ATCACCTCAAAAACCTAAAC TAGTGGGTCAGTGTGGTTAG | GA | 229 | 48 | No | Isagi and Suhandono 1997 |
| QM55GA | ATTTGATATTTGATGTTGTTT TGATGTAACATTGCTTAGAGT | GA | 306 | 47 | No | Isagi and Suhandono 1997 |
| QM63-2M3 | TTATTGTATATGCTGGTTCT GATTGAATAGGAAAAGAGTG | TG | 155 | 47 | No | Isagi and Suhandono 1997 |
| QM57-3M | TGAGGAGGTTGGTGGAGAA ATGTGGAGGGCTAAGATTT | TGG | 235 | 53 | No | Isagi and Suhandono 1997 |
| QM58TGT | GGTCAGTGTATTTTGTTGGT AAATGTATTTTGCTTGCTCA | CAA | 212 | 51 | No | Isagi and Suhandono 1997 |
| GOT021 | AGAAAGTTCCAGGGAAAGCA CTTCGTCCCCAGTTGAATGT | AT | 111-128 | 56 | Yes | Durand *et al*. 2010 |
| FIR026 | CTTCATGCACCAATTCCTCA GGCCATGTATGTGTGCAAAA | TC | 208-217 | 56 | Yes | Durand *et al*. 2010 |
| QmC00716 | AAGAGAACCCATTCCATCCCTGA GTTTCCCGAACAGTGGTTTCTTGA | TC | 261-287 | 56 | No | Ueno *et al*.2008 |
| POR017 | CCCATATCCCTCTACGAAAGAA CTGGAGATGACATAGTGTCTCAAA | CT | 140-169 | 54 | Yes | Durand *et al*. 2010 |
| FIR015 | ACCCTAAAACCCCAATCACC  CGGATCTTCGGCTATTCTTG | AC | 128-138 | 56 | Yes | Durand *et al*.2010 |
| QmC00932 | AGGCTCAAAACAAAACCAAACCG GTTTCCCCTTTCCCATAATCAAACCCT | TC | 247-260 | 54 | Yes | Ueno *et al*.2008 |
| DN950446 | TCTCTTTCTCCGTCCATTATCGC GTTTCTCCACAGACCCCATTTCC | AG | 155-185 | 56 | No | Ueno and Tsumura 2008 |
| WAG068 | TCTGCAACAAAACCAAAACAC CGGAGGAGAGAGTCAGCAAC | AG | 165-195 | 56 | Yes | Durand *et al*.2010 |
| PIE271 | CACACTCACCAACCCTACCC GTGCGGTTGTAGACGGAGAT | TC | 197-247 | 56 | No | Durand *et al*.2010 |
| QmC02052 | CACACCCAGATCCACAAAACTCC GTTTGCCTCTACGGTCTCCCTCTT | AG | 250-300 | 56 | Yes | Ueno *et al*.2008 |
| GOT011 | CCCCACCGTCTACTCTCAAA GCGTTCACCACGTCCATAAT | TC | 197-255 | 56 | Yes | Durand *et al*. 2010 |
| WAG066 | AACCTGTTTGGCTTCGTGTG AACAAAAGATTGGGAGGTGC | AG | 128-244 | 56 | Yes | Durand *et al*. 2010 |
| QrZAG30 | TGCTCCGTCATAATCTTGCTCTGA GCAATCCTATCATGCACATGCACAT | GA | 160-208 | 56 | No | Kampfer *et al*.1998 |
| QpZAG9 | GCAATTACAGGCTAGGCTGG GTCTGGACCTAGCCCTCATG | TG | 220-274 | 56 | No | Steinkellner *et al*. 1997 |
| QrZAG112 | TTCTTGCTTTGGTGCGCG GTGGTCAGAG ACTCGGTAAGTATTC | GA | 98-116 | 56 | No | Kampfer *et al*. 1998 |
| GOT012 | TGATGATCCCAAACCACAAA AAGGCTGCAGGACTTTTCAA | GT | 204-254 | 56 | No | Durand *et al*. 2010 |
| GOT040 | AAGGCACTCGTCGCTTTCTA ACCGATTTGAAGCTCGAGAA | TC | 242-298 | 56 | No | Durand *et al*. 2010 |
| PIE163 | GAGAGGCATGTGGAACCAAG CAAGCATAGGTGGTGGAACC | TC | 230-264 | 56 | No | Durand *et al*. 2010 |
| POR025 | CACACAAACCCATATGATCTGAA TCTCTTTCGATCCCTTCTGC | AG | 105-145 | 56 | No | Durand *et al*. 2010 |
| QpZAG16 | CTTCACTGGCTTTTCCTCCT TGAAGCCCTTGTCAACATGC | TC | 131-189 | 56 | No | Steinkellner *et al*. 1997 |
| QpZAG110 | GGAGGCTTCCTTCAACCTACT GATCTCTTGTGTGCTGTATTT | AG | 193-267 | 56 | No | Steinkellner *et al*. 1997 |
| QmC02241 | TCAGTGACCACACGTCACCTCTC GTTTCTTGGCCATGTTTTGATGG | GA/TC | 169-211 | 56 | No | Ueno *et al*. 2008 |

**Cited references:**

Durand, J., Bodénès, C., Chancerel, E., Frigerio, J. M., Vendramin, G., Sebastiani, F., Buonamici, A., Gailing, O., Koelewijn, H. P., Villani, F., and Mattioni, C. (2010). A fast and cost-effective approach to develop and map EST-SSR markers: Oak as a case study. *BMC genomics*, *11*, 1-13. https://doi.org/10.1186/1471-2164-11-570

Huang, G., Hong, L., Ye, W., Shen, H., Cao, H., and Xiao, W. (2009). Isolation and characterization of polymorphic microsatellite loci in *Castanopsis chinensis* Hance (Fagaceae). *Conservation genetics*, *10*, 1069-1071. https://doi.org/10.1007/s10592-008-9690-2

Isagi, Y., and Suhandono, S. (1997). PCR primers amplifying microsatellite loci of *Quercus myrsinifolia* Blume and their conservation between oak species. *Molecular Ecology*, *6*(9), 897-899. https://doi.org/10.1046/j.1365-294X.1997.d01-218.x

Kampfer, S., Lexer, C., Glössl, J., and Steinkellner, H. (1998). Brief report characterization of (GA) n microsatellite loci from *Quercus robur*. *Hereditas*, *129*(2), 183-186.

Marinoni, D., Akkak, A., Bounous, G., Edwards, K. J., and Botta, R. (2003). Development and characterization of microsatellite markers in *Castanea sativa* (Mill.). *Molecular Breeding*, *11*(2), 127-136. https://doi.org/10.1023/A:1022456013692

Sebastiani, F., Carnevale, S., and Vendramin, G. G. (2004). A new set of mono‐and dinucleotide chloroplast microsatellites in Fagaceae. *Molecular Ecology Notes*, *4*(2), 259-261. https://doi.org/10.1111/j.1471-8286.2004.00635.x

Shi, Y. S., Zhang, J., Jiang, K., Cui, M. Y., and Li, Y. Y. (2011). Development and characterization of polymorphic microsatellite markers in *Castanopsis sclerophylla* (Fagaceae). *American Journal of Botany*, *98*(2), e19-e21. https://doi.org/10.3732/ajb.1000400

Steinkellner, H., Fluch, S., Turetschek, E., Lexer, C., Streiff, R., Kremer, A., Burg, K., and Glössl, J. (1997). Identification and characterization of (GA/CT) n-microsatellite loci from *Quercus petraea*. *Plant Molecular Biology*, *33*(6), 1093-1096. https://doi.org/10.1023/A:1005736722794

Tong, X., Xu, N. N., Li, L., and Chen, X. Y. (2012). Development and characterization of polymorphic microsatellite markers in *Cyclobalanopsis glauca* (Fagaceae). *American Journal of Botany*, *99*(3), e120-e122. https://doi.org/10.3732/ajb.1100448

Ueno, S., and Tsumura, Y. (2008). Development of ten microsatellite markers for *Quercus mongolica* var. *crispula* by database mining. *Conservation Genetics*, *9*(4), 1083-1085. https://doi.org/10.1007/s10592-007-9462-4

Ueno, S., Yoshimaru, H., Kawahara, T., and Yamamoto, S. (2003). A further six microsatellite markers for *Castanopsis cuspidata* var. *sieboldii* Nakai. *Conservation* *Genetics*, *4*(6), 813-815. https://doi.org/10.1023/B:COGE.0000006120.99339.10

Ueno, S., Taguchi, Y., and Tsumura, Y. (2008). Microsatellite markers derived from *Quercus mongolica* var. *crispula* (Fagaceae) inner bark expressed sequence tags. *Genes & Genetic Systems*, *83*(2), 179-187. https://doi.org/10.1266/ggs.83.179

Waikham, P., Thongkumkoon, P., Chomdej, S., Liu, A., and Wangpakapattanawong, P. (2018). Development of 13 microsatellite markers for *Castanopsis tribuloides* (Fagaceae) using next-generation sequencing. *Molecular Biology Reports*, *45*(5), 27-30. https://doi.org/10.1007/s11033-017-4137-9

**Table S3** Genetic diversity for pure individuals of the five Fagaceae species estimated based on nuclear microsatellite (nSSR) data.

| Species | *N*_A_ | *N*_E_ | *I* | *H*_O_ | *H*_E_ | *uH*_E_ |
| --- | --- | --- | --- | --- | --- | --- |
| *Q. glauca* | 6.80 | 2.90 | 1.11 | 0.59 | 0.54 | 0.54 |
| *Q. multinervis* | 7.10 | 3.38 | 1.16 | 0.60 | 0.54 | 0.54 |
| *C. tibetana* | 7.70 | 3.00 | 1.21 | 0.58 | 0.57 | 0.58 |
| *C. faberi* | 6.89 | 4.27 | 1.36 | 0.54 | 0.60 | 0.62 |
| *C. fargesii* | 6.40 | 3.32 | 1.26 | 0.59 | 0.62 | 0.64 |
| All species | 6.98 | 3.37 | 1.22 | 0.58 | 0.57 | 0.58 |

Note: *N*_A_, number of different alleles; *N*_E_, number of effective alleles; *I*, Shannon’s information index; *H*_O_, observed heterozygosity; *H*_E_, expected heterozygosity; *uH*_E_, unbiased expected heterozygosity. Indices have statistically significant different values among the five Fagaceae species (*P* < 0.001).

**Table S4** Genetic diversity for all individuals of the five Fagaceae species estimated based on nuclear microsatellite (nSSR) data.

| Species | *N*_A_ | *N*_E_ | *I* | *H*_O_ | *H*_E_ | *uH*_E_ |
| --- | --- | --- | --- | --- | --- | --- |
| *Q. glauca* | 7.70 | 3.13 | 1.18 | 0.58 | 0.55 | 0.56 |
| *Q. multinervis* | 8.40 | 3.62 | 1.23 | 0.60 | 0.56 | 0.56 |
| *C. tibetana* | 8.80 | 3.34 | 1.31 | 0.58 | 0.60 | 0.60 |
| *C. faberi* | 7.24 | 4.36 | 1.39 | 0.55 | 0.61 | 0.62 |
| *C. fargesii* | 6.90 | 3.30 | 1.27 | 0.60 | 0.61 | 0.63 |
| All species | 7.81 | 3.45 | 1.28 | 0.58 | 0.59 | 0.59 |

Note: *N*_A_, number of different alleles; *N*_E_, number of effective alleles; *I*, Shannon’s information index; *H*_O_, observed heterozygosity; *H*_E_, expected heterozygosity; *uH*_E_, unbiased expected heterozygosity. Indices have statistically significant different values among the five Fagaceae species (*P* < 0.001).

**Table S5** Analysis of molecular variance (AMOVA) for all individuals of the five Fagaceae species based on nuclear microsatellite (nSSR) data.

| Source of variation | *df* | SS | VC | V% | *F*_ST_ |
| --- | --- | --- | --- | --- | --- |
| ***Q. glauca*** |  |  |  |  |  |
| Among populations | 7 | 146.04 | 0.36 | 11.67 | 0.12 |
| Within populations | 406 | 1092.43 | 2.69 | 88.33 |  |
| ***Q. multinervis*** |  |  |  |  |  |
| Among populations | 2 | 38.22 | 0.24 | 7.08 | 0.07 |
| Within populations | 235 | 749.89 | 3.19 | 92.92 |  |
| ***C. tibetana*** |  |  |  |  |  |
| Among populations | 6 | 39.88 | 0.13 | 5.00 | 0.05 |
| Within populations | 215 | 545.90 | 2.54 | 95.00 |  |
| ***C. faberi*** |  |  |  |  |  |
| Among populations | 6 | 27.98 | 0.08 | 3.08 | 0.03 |
| Within populations | 197 | 479.36 | 2.43 | 96.92 |  |
| ***C. fargesii*** |  |  |  |  |  |
| Among populations | 3 | 11.39 | 0.05 | 1.78 | 0.02 |
| Within populations | 120 | 297.74 | 2.48 | 98.22 |  |
| **All species** |  |  |  |  |  |
| Among species | 4 | 914.73 | 0.92 | 22.32 |  |
| Among populations within species | 24 | 299.18 | 0.24 | 5.92 | 0.28 |
| Within populations | 1173 | 3473.53 | 2.96 | 71.76 |  |

Note: *df*, degrees of freedom; SS, sum of squares; VC, variance component; V%, percentage of variation; *F*_ST_, differentiation among populations. Significance tests (1000 permutations) showed all fixation indices were significant (*P* < 0.001).

**Table S6** One-way analysis of variance (one-way ANOVA) for seven traditional leaf morphological traits for pure individuals of the five Fagaceae species.

| Traditional leaf morphological traits | Difference source | *df* | Mean square | *F* | *P* |
| --- | --- | --- | --- | --- | --- |
| Leaf length (LL) | Among species | 4 | 9677.51 | 1941.18 | < 0.01 |
|  | Within specie | 2605 | 4.99 |  |  |
|  | Sum | 2609 |  |  |  |
| Petiole length (PL) | Among species | 4 | 212.76 | 1239.39 | < 0.01 |
|  | Within specie | 2605 | 0.17 |  |  |
|  | Sum | 2609 |  |  |  |
| Leaf width (LW) | Among species | 4 | 481.70 | 2138.94 | < 0.01 |
|  | Within specie | 2605 | 0.23 |  |  |
|  | Sum | 2609 |  |  |  |
| Length of lamina from base to widest point (WP) | Among species | 4 | 3156.59 | 1412.19 | < 0.01 |
|  | Within specie | 2605 | 2.24 |  |  |
|  | Sum | 2609 |  |  |  |
| Leaf mass (LM) | Among species | 4 | 380.71 | 2041.78 | < 0.01 |
|  | Within specie | 2605 | 0.19 |  |  |
|  | Sum | 2609 |  |  |  |
| Leaf area (LA) | Among species | 4 | 897,866.32 | 2215.91 | < 0.01 |
|  | Within specie | 2605 | 405.19 |  |  |
|  | Sum | 2609 |  |  |  |
| Specific leaf area (SLA) | Among species | 4 | 34,505.95 | 417.27 | < 0.01 |
|  | Within specie | 2605 | 82.69 |  |  |
|  | Sum | 2609 |  |  |  |

Note: *df*, degrees of freedom; *F*, *F* values; *P* value indicates statistical significance.

**Table S7** One-way analysis of variance (one-way ANOVA) for seven traditional leaf morphological traits for all individuals of the five Fagaceae species.

| Traditional leaf morphological traits | Difference source | *df* | Mean square | *F* | *P* |
| --- | --- | --- | --- | --- | --- |
| Leaf length (LL) | Among species | 4 | 12,533.68 | 2335.22 | < 0.01 |
|  | Within specie | 3000 | 5.37 |  |  |
|  | Sum | 3004 |  |  |  |
| Petiole length (PL) | Among species | 4 | 239.27 | 1366.68 | < 0.01 |
|  | Within specie | 3000 | 0.18 |  |  |
|  | Sum | 3004 |  |  |  |
| Leaf width (LW) | Among species | 4 | 623.11 | 2663.45 | < 0.01 |
|  | Within specie | 3000 | 0.23 |  |  |
|  | Sum | 3004 |  |  |  |
| Length of lamina from base to widest point (WP) | Among species | 4 | 4157.57 | 1777.50 | < 0.01 |
|  | Within specie | 3000 | 2.34 |  |  |
|  | Sum | 3004 |  |  |  |
| Leaf mass (LM) | Among species | 4 | 503.19 | 2451.33 | < 0.01 |
|  | Within specie | 3000 | 0.21 |  |  |
|  | Sum | 3004 |  |  |  |
| Leaf area (LA) | Among species | 4 | 1,181,772.57 | 2695.20 | < 0.01 |
|  | Within specie | 3000 | 438.47 |  |  |
|  | Sum | 3004 |  |  |  |
| Specific leaf area (SLA) | Among species | 4 | 40,697.73 | 502.20 | < 0.01 |
|  | Within specie | 3000 | 81.04 |  |  |
|  | Sum | 3004 |  |  |  |

Note: *df*, degrees of freedom; *F*, *F* values; *P* value indicates statistical significance.

**Table S8** Means and standard deviations (SD) of seven traditional leaf morphological traits for all individuals of the five Fagaceae species and coefficient of variation (CV) for each trait.

| Traditional leaf morphological traits | *Q. glauca* | *Q. multinervis* | *C. tibetana* | *C. faberi* | *C. fargesii* | CV (%) |
| --- | --- | --- | --- | --- | --- | --- |
| Leaf length (LL) (cm) | 12.26 ± 1.85 | 13.21 ± 2.02 | 22.38 ± 3.75 | 11.45 ± 1.76 | 10.25 ± 1.59 | 33.61 |
| Petiole length (PL) (cm) | 2.13 ± 0.51 | 1.71 ± 0.40 | 2.14 ± 0.43 | 0.80 ± 0.30 | 0.80 ± 0.20 | 41.56 |
| Leaf width (LW) (cm) | 2.47 ± 0.46 | 2.38 ± 0.41 | 4.46 ± 0.76 | 1.94 ± 0.31 | 1.60 ± 0.24 | 39.04 |
| Length of lamina from base to widest point (WP) (cm) | 6.08 ± 1.19 | 6.34 ± 1.28 | 11.60 ± 2.61 | 4.91 ± 1.09 | 4.71 ± 0.78 | 41.18 |
| Leaf mass (LM) (g) | 0.68 ± 0.24 | 0.60 ± 0.19 | 2.65 ± 0.97 | 0.44 ± 0.15 | 0.31 ± 0.09 | 98.45 |
| Leaf area (LA) (cm^2^) | 40.85 ± 12.47 | 42.30 ± 12.27 | 137.72 ± 42.74 | 30.59 ± 8.82 | 22.74 ± 6.11 | 80.91 |
| Specific leaf area (SLA) (cm^2^·g^-1^) | 61.50 ± 8.37 | 72.02 ± 10.33 | 53.34 ± 7.91 | 70.17 ± 9.25 | 75.12 ± 9.64 | 17.89 |

**Table S9** Statistical parameters of the log-log linear relations between leaf area (LA) and leaf mass (LM) for all individuals of the five Fagaceae species.

|  | *Q. glauca* | *Q. multinervis* | *C. tibetana* | *C. faberi* | *C. fargesii* |
| --- | --- | --- | --- | --- | --- |
| α | 0.80 | 0.81 | 0.80 | 0.80 | 0.84 |
| log β | 1.75 | 1.81 | 1.80 | 1.77 | 1.79 |
| 95% CI | (0.77, 0.82) | (0.78, 0.84) | (0.77, 0.83) | (0.77, 0.84) | (0.79, 0.88) |
| *R*^2^ | 0.84 | 0.80 | 0.83 | 0.81 | 0.82 |
| *P* | < 0.001 | < 0.001 | < 0.001 | < 0.001 | < 0.001 |

Note: α, the slope of the log_10_-transformed LA vs. LM regression curve; β, the elevation of the log_10_-transformed LA vs. LM regression curve; CI, confidence intervals; *R*^2^, coefficient of determination; *P* value indicates statistical significance.
